# A chemo-mechanical model of endoderm movements driving elongation of the amniote hindgut

**DOI:** 10.1242/dev.202010

**Published:** 2023-11-16

**Authors:** Panagiotis Oikonomou, Helena C. Cirne, Nandan L. Nerurkar

**Affiliations:** Department of Biomedical Engineering, Columbia University, 351 Engineering Terrace, 1210 Amsterdam Avenue, New York, NY 10027, USA

**Keywords:** Morphogenesis, Chemo-mechanical model, Hindgut, Chick, Endoderm

## Abstract

Although mechanical and biochemical descriptions of development are each essential, integration of upstream morphogenic cues with downstream tissue mechanics remains understudied during vertebrate morphogenesis. Here, we developed a two-dimensional chemo-mechanical model to investigate how mechanical properties of the endoderm and transport properties of fibroblast growth factor (FGF) regulate avian hindgut morphogenesis in a coordinated manner. Posterior endoderm cells convert a gradient of FGF ligands into a contractile force gradient, leading to a force imbalance that drives collective cell movements that elongate the forming hindgut tube. We formulated a 2D reaction-diffusion-advection model describing the formation of an FGF protein gradient as a result of posterior displacement of cells transcribing unstable *Fgf8* mRNA during axis elongation, coupled with translation, diffusion and degradation of FGF protein. The endoderm was modeled as an active viscous fluid that generates contractile stresses in proportion to FGF concentration. With parameter values constrained by experimental data, the model replicates key aspects of hindgut morphogenesis, suggests that graded isotropic contraction is sufficient to generate large anisotropic cell movements, and provides new insight into how chemo-mechanical coupling across the mesoderm and endoderm coordinates hindgut elongation with axis elongation.

## Teaser

**Summary:** Formulation of a mathematical model to investigate the interplay between morphogen gradients and tissue mechanics in regulating the collective cell movements that drive hindgut morphogenesis in the chick embryo.

## INTRODUCTION

Embryogenesis requires coordination of cell fate specification with profound physical transformations during development. Accordingly, there has been extensive recent work developing a mechanically motivated framework for studying morphogenesis (Collinet and Lecuit, 2021; Goodwin and Nelson, 2021; Valet et al., 2022). These efforts have led to advances in understanding many aspects of development, including how forces deform tissues to create precise morphological outcomes (Garcia et al., 2019; Heer et al., 2017; Nelson and Gleghorn, 2012; Savin et al., 2011), how the subcellular machinery that enacts those forces is regulated (Kasza et al., 2014; Lecuit and Yap, 2015), and how forces can themselves regulate a range of cell behaviors (Barriga et al., 2018; Desprat et al., 2008; Godard et al., 2020; Pan et al., 2016). Despite these advances, mechanical and biochemical descriptions of vertebrate development have often been invoked as orthogonal to one another, with physical mechanisms, such as buckling, often viewed as mutually exclusive with those regulated by diffusible signals, such as Turing patterns (Shyer et al., 2013; Walton et al., 2015). As a result, the integration of biochemical cues with a mechanical description of morphogenesis has been somewhat limited (Durel and Nerurkar, 2020). In particular, it remains unclear how physical properties of embryonic tissue are coordinated with transport properties of morphogenic signals to guide vertebrate embryogenesis. The present work employs a mathematical model to study these links during morphogenesis of the avian hindgut.

**Fig. 1.**
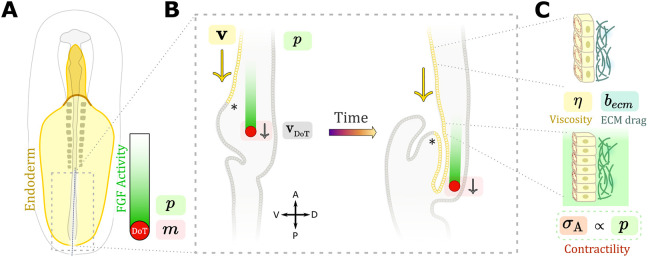
**Schematic overview of avian hindgut morphogenesis.** (A) A chick embryo (HH stage 10, approximately 1.5 days of development) with the endodermal epithelium shown in yellow. At this stage, an antero-posterior gradient of FGF protein (green) has already been established, owing to cells in the PSM that are shed from the domain of active transcription (DoT, red) where self-renewing cells actively transcribe FGF genes. (B) Sagittal view of the tailbud region illustrating hindgut tube formation. The endodermal sheet (yellow) and the DoT (red) move at different rates (***v*** versus v_DoT_). (C) The mechanical properties of the endoderm affect its subsequent movements via activation of isotropic contraction as a result of FGF signaling. Anatomical axes: A, anterior; D, dorsal; P, posterior; V, ventral. Asterisk in B marks the caudal intestinal portal. *m* is the FGF mRNA concentration, *p* the FGF protein concentration, *η* the endoderm viscosity, *b*_*ecm*_ the viscous drag coefficient from ECM interactions, and *σ*_*A*_ the active stress from acto-myosin contractility.

The gut tube is an endodermally derived epithelial structure that gives rise to the inner lining of the respiratory and gastrointestinal tracts (Zorn and Wells, 2009). The posterior-most segment of the gut tube, the hindgut, forms via collective endoderm cell movements that outpace axis elongation (Nerurkar et al., 2019). This mismatch in elongation rates is accommodated by dorso-ventral inversion of the endoderm, leading to progressive folding and elongation of the hindgut tube during outgrowth of the tailbud (Fig. 1A,B). As such, these collective cell movements are a central driver of hindgut formation, and it is crucially important that they be coordinated with axis elongation. This coordination is achieved by dependence of both events on the same molecular cue: a long-range gradient of fibroblast growth factor (FGF) ligands (Bénazéraf et al., 2010; Dubrulle and Pourquié, 2004; Nerurkar et al., 2019). *Fgf4* and *Fgf8* are expressed in a posterior-to-anterior gradient adjacent to the endoderm in the tailbud mesenchyme and presomitic mesoderm (PSM) (Boulet and Capecchi, 2012; Crossley and Martin, 1995; Dubrulle et al., 2001). Endoderm cells convert this FGF protein gradient into a gradient of acto-myosin activity, producing a force imbalance that pulls cells toward the tail end of the embryo (Fig. 1C) (Nerurkar et al., 2019). This generates a positive-feedback loop whereby anterior cells exposed to low FGF levels are displaced passively to higher FGF levels, resulting in a progressive recruitment of contracting cells toward the forming hindgut. This is counteracted by posterior displacement of the FGF gradient itself as the embryo elongates (Bénazéraf et al., 2010; Boulet and Capecchi, 2012; Dubrulle et al., 2001; Sawada et al., 2001).

Classically, morphogen gradients have been thought to arise as a result of diffusion of protein from a localized source across a field of cells (Crick, 1970; Driever and Nüsslein-Volhard, 1988). Although this is the case in many contexts (Müller et al., 2012; Simsek and Özbudak, 2018; Wolpert, 1969; Yu et al., 2009), morphogen gradients can also form by advection, whereby transport due to tissue movements predominates (Ibañes et al., 2006; Pfeiffer et al., 2000; Stapornwongkul and Vincent, 2021). The posterior FGF gradient, for example, forms by posterior-ward displacement of a restricted population of self-renewing cells that actively transcribe *Fgf8*; cells are shed from this domain as axis elongation proceeds, retaining unstable transcripts that are degraded over time (Dubrulle and Pourquié, 2004). As a result, anterior cells retain fewer transcripts than posterior cells. When coupled with translation and diffusion of the resulting FGF protein, this results in the formation of an anisotropic FGF protein gradient that is accentuated along the antero-posterior axis (Dubrulle and Pourquié, 2004).

The present study formulates a 2D chemo-mechanical model to investigate how tissue mechanics and spatial control of cell behavior by diffusible signals coordinately regulate cell movements in the hindgut endoderm. We first constructed a 2D model of FGF gradient formation to investigate how FGF transport properties interact with the rate of axis elongation to control the shape and extent of the FGF protein gradient. Next, we developed a continuum model of the endoderm as an active fluid, examining how FGF concentration-dependent active tension regulates tissue flows during extension of the hindgut. The model replicates several experimentally observed behaviors of the posterior chick endoderm, and provides evidence that graded, but isotropic (equal in all directions) cell contractility is sufficient to generate highly anisotropic (directionally biased) collective movements in the absence of any other asymmetries. Finally, exploring the parameter space of the model provides new insight into the efficiency and evolvability of morphogenic mechanisms, and how chemo-mechanical coupling across the mesoderm and endoderm coordinates hindgut elongation with outgrowth of the tailbud.

## RESULTS

### 2D mRNA decay model quantitatively replicates embryonic FGF activity gradient in the endoderm

In order to study the chemo-mechanical basis of cell movements driving hindgut formation, we first formulated a 2D model describing the establishment and maintenance of an anisotropic, 2D FGF protein gradient. To model gradient formation by progressive posterior displacement of this domain of active transcription (DoT) (Fig. 1) (Dubrulle and Pourquié, 2004), we formulated a 2D reaction-diffusion-advection framework of coupled partial differential equations that builds on a previous 1D model (Harrison et al., 2011). We modeled transport of two species, *Fgf8* mRNA (*m*) and the FGF8 protein (*p*). Production of *m* by transcription at a rate *r*_*m*_ was restricted to the DoT, moving posteriorly with velocity *v*_DoT_ (Fig. 1A,B). Transcripts left in the wake of the DoT were specified to degrade in proportion to concentration *m* at a rate given by *μ*_*m*_. FGF protein *p* was modeled as being translated in proportion to the concentration *m* at a rate *β*_*p*_, and degraded at a rate proportional to protein concentration *p*, given by *μ*_*p*_. Finally, whereas mRNA remains restricted within cells, FGF protein *p* diffused with a diffusion coefficient *D*_*p*_. When formulated in a reference frame that moves with the DoT, the associated mass balance results in a pair of coupled partial differential equations (see Materials and Methods and supplementary Materials and Methods for further details):
(1)

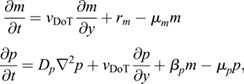
where *t* is time. The model was de-dimensionalized by defining scaled variables:
(2)

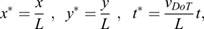
where *L* represents antero-posterior extent of the endoderm at the start of simulations. Substituting Eqn 2 into Eqn 1 produces the dimensionless form:
(3)

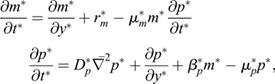
where the six model parameters reduce to four dimensionless ones:
(4)

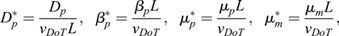


where 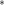 denotes unit-less parameters and variables, and 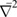 is the Laplacian with respect to scaled spatial coordinates.

**Fig. 2.**
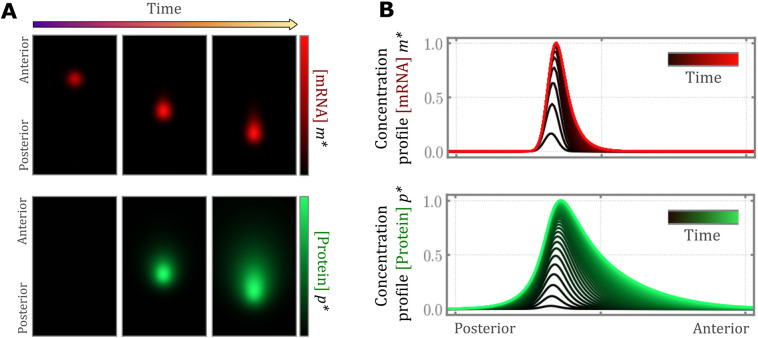
**Simulation of the mRNA decay model and corresponding mRNA and protein concentration profiles.** (A) 2D concentration profiles of mRNA (top, red) and protein (bottom, green) as visualized in the fixed/laboratory frame of reference as the embryo elongates and the DoT moves posteriorly. (B) The temporal evolution of the concentration profiles for mRNA and protein along the midline of the embryo in the moving frame of reference. Line color indicates temporal progression from the initial time point (black) to the steady state mRNA (red) and protein (green) concentrations for constant time steps between each successive curve.

**Fig. 3.**
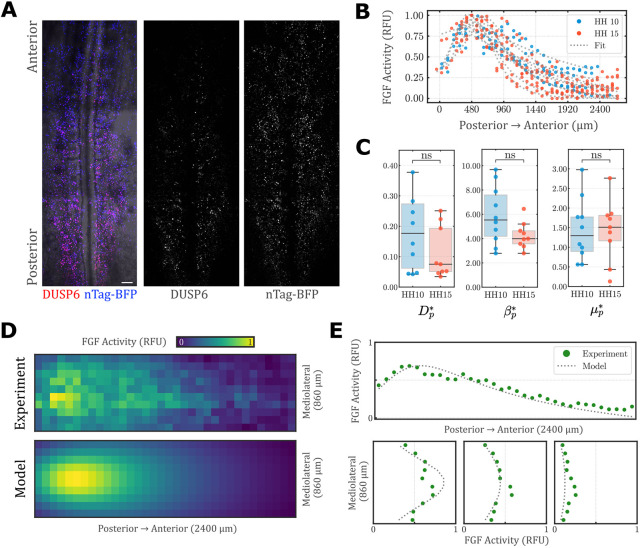
**Measurement of FGF pathway activity *in vivo*.** (A) Representative ventral view of an HH stage 15 embryo following co-electroporation of the posterior endoderm with the FGF-responsive *Dusp6*-mScarlet reporter and the ubiquitous reporter nTag-BFP (control). Scale bar: 100*μ*m. (B,C) Normalized medial *Dusp6* reporter intensity varies with antero-posterior position at stages HH 10 (blue) and HH 15 (red); dotted lines indicate the model fit to experimental data used to establish physiological values of the model parameters 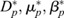 (Table 1) (quantified in C) for HH 10 (*n*=9) and HH 15 (*n*=10); ns, no significant difference by two-sided Mann–Whitney–Wilcoxon Test. No statistical method was used to determine sample size *a priori*. The box edges represent the Q1 to Q3 quartile values, the horizontal line represents the median (Q2) and the whiskers extend to show the range of the data, no more than 1.5× IQR (IQR=Q3-Q1) from the edges of the box, ending at the farthest data point within that interval (from pandas documentation: https://pandas.pydata.org/docs/reference/api/pandas.DataFrame.boxplot.html). (D) Comparison of 2D representation of FGF activity from averaged *Dusp6*-mScarlet/nTag-BFP electroporation and model-simulated FGF activity. (E) Comparison of FGF activity on virtual sagittal (top) and transverse (bottom) slices. RFU, relative fluorescent units, determined by normalizing the intensity of the *Dusp6*-mScarlet reporter to nTag-BFP control.

Simulations based on the solution of Eqn 3 revealed the development of a compact, anisotropic gradient of mRNA concentration 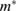 that is progressively displaced posteriorly with axis elongation (Fig. 2A, Movie 1). This resulted in a traveling, long-range gradient of FGF protein 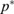 that extends beyond the mRNA gradient, and has a more pronounced anisotropy (Fig. 2). When solved in a frame of reference traveling with the domain of transcription, the model smoothly approaches a stable steady state solution over time (Fig. 2B, Fig. S1). To test whether the FGF gradient does in fact stabilize prior to the onset of hindgut morphogenesis, FGF activity in the endoderm was quantified using a reporter in which the FGF-responsive *Dusp6* promoter is placed upstream of a fluorescent readout (Fig. 3A) (Nerurkar et al., 2019). Comparison of the antero-posterior shape of the FGF activity gradient between Hamburger Hamilton (HH) stage 10 (Hamburger and Hamilton, 1951), prior to the onset of hindgut formation, and HH 15, when hindgut formation is already underway, revealed no qualitative difference between these stages (Fig. 3B). To test this quantitatively, *Dusp6* reporter activity was analyzed in terms of model parameters describing FGF protein transport (see supplementary Materials and Methods for further details). We observed no difference in diffusion (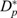), protein decay rate (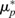) or protein translation rate (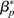) between stages HH 10 and 15 (Fig. 3C). These results offer validation, confirming the model prediction that the FGF gradient approaches a steady-state solution, after which axis elongation results in a posterior shift in the FGF gradient without changing its shape. Moreover, the quantification of model parameters from experimental data provides an important physiologic baseline (Table 1) from which parametric sweeps can be conducted. Therefore, the model captures the basic shape and extent of the *in vivo* FGF activity gradient using model parameters constrained by experimental data (Fig. 3).

**Table 1.**
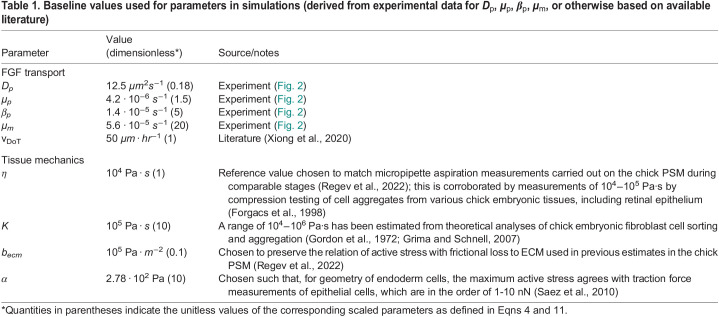
Baseline values used for parameters in simulations (derived from experimental data for *D*_***p***_, *μ*_***p***_, *β*_***p***_, *μ*_***m***_, or otherwise based on available literature)

### mRNA and protein kinetics differentially influence morphogen gradient shape

**Fig. 4.**
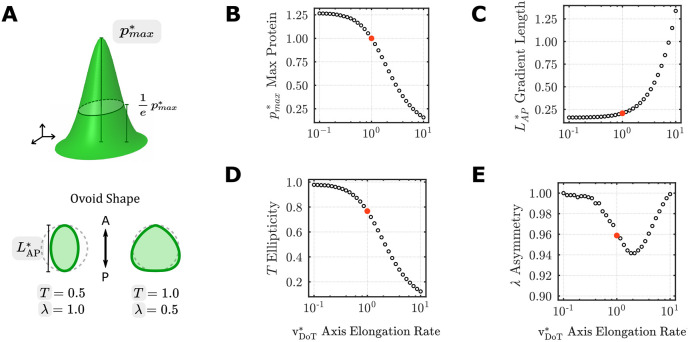
**Effects of axis elongation rate on 2D FGF gradient shape.** (A) Schematic of output metrics, including maximal protein level p_max_^*^ (top), and anteroposterior length (*L*_AP_^*^), ellipticity (T) and asymmetry (λ) (bottom). (B-E) Parametric sweeps on 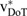; red circle denotes the experimentally measured axis elongation rate in wild-type chick embryos (Table 1). Protein levels were normalized to the maximum protein value from the physiological set of parameters.

With baseline physiologic values in place for all transport parameters (Table 1), we next conducted parametric sweeps to investigate how each property influences gradient shape. To characterize the resulting 2D FGF concentration profiles, isoline contours corresponding to 1/*e* of the maximal protein concentration (the distance at which 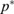 diminishes to ∼37% of its peak) were quantified with respect to their ellipticity (*T*) and asymmetry (*λ*) (Fig. 4A) (Baker, 2002). *T*=*λ*=1 indicates a radially symmetric (isotropic) protein concentration profile, *T*<1 indicates antero-posterior elongation, and *λ*<1 indicates posteriorly biased asymmetry along the antero-posterior axis (Fig. 4A). In addition to gradient shape, we quantified the maximum protein concentration (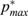) and scaled length of the gradient (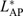).

Given that scaling of the transport equations resulted in absorption of v_DoT_ into the scaled parameters (Eqn 4), the effects of changes in this key parameter were first isolated to simplify the interpretation of subsequent parametric sweeps. Increasing 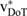 reduced 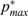, owing to a constant rate of transcription and translation resulting in similar amounts of total FGF protein distributed across a broader domain as the embryo elongates posteriorly at higher speeds (Fig. 4B). 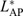, indicating the antero-posterior length over which the FGF gradient acts, varied directly with 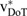 (Fig. 4C), confirming the central role of axis elongation in the establishment of the long-range FGF gradient (Dubrulle et al., 2001; Harrison et al., 2011). This effect was highly anisotropic, as seen by a corresponding decrease in ellipticity (T) (Fig. 4D) and little change in the medio-lateral extent of the gradient (Fig. S2). A small, but nonlinear, dependence of asymmetry *λ* on 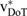 was observed: *λ* changed by less than 10% when 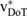 was varied across two orders of magnitude (Fig. 4E).

**Fig. 5.**
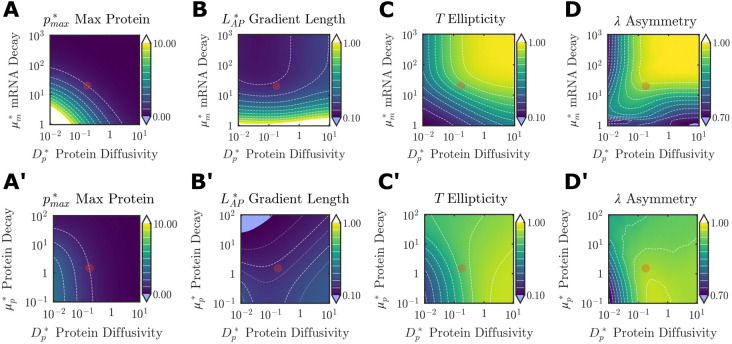
**Parametric sweeps on FGF transport properties.** Output metrics describing 2D FGF protein gradient shape across parametric sweeps on mRNA decay 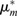 and protein diffusivity 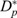 (A-D), as well as protein decay 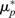 and protein diffusivity 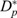 (A′-D′). Red circle denotes the baseline values quantified experimentally (Fig. 2C). White dashed lines are isolines at 10% intervals of the color bar range; in B′, gray isolines are at 5% intervals. Protein levels were normalized to the maximum protein value from the physiological set of parameters.

We next considered how shape of the FGF gradient depends on the scaled mRNA decay rate (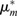) and protein diffusion (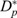). An increase in 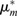 indicates that the system is dominated by the instability of FGF transcripts rather than the rate of axis elongation, whereas an increase in 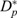 favors protein to diffuse from its site of translation over the effects of axis elongation. Both parameters strongly influence overall features of the FGF gradient (Fig. 5A-D). 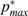 is highest when both parameters are small (Fig. 5A), reflecting a long lifetime for FGF transcripts and protein that accumulates locally with limited diffusion from the site of translation. Experimentally based approximations of 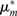 and 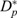 (Fig. 3C, Table 1) fall well outside the range of maximal protein concentration, consistent with the importance of the spatial distribution of FGF rather than its absolute levels for axis elongation (Bénazéraf et al., 2010) and hindgut morphogenesis (Nerurkar et al., 2019) (Fig. 5A). The antero-posterior extent of the FGF gradient 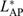 depends nonlinearly on 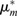, with relatively little change as 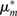 increases beyond baseline values approximated in the chick embryo (Fig. 5B). Interestingly, although 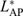 does increase in response to increased diffusion 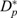, it is far more sensitive to changes in 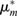 (Fig. 5B). Finally, in the limit of increasing 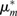 and 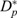, the effects of axis elongation rate were minimized in favor of highly unstable transcripts that are translated into proteins that rapidly diffuse from their source, thereby approaching the case of radially symmetric diffusion from a static source (Fig. 5C,D). This further supports the central role of axis elongation in anisotropic gradient formation. 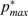 and 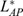 were generally less sensitive to instability at the protein level (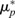) than at the mRNA level (Fig. 5A′,B′). Notably, although protein diffusivity 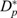 did not have a strong influence on the antero-posterior extent of the FGF gradient across a broad range of mRNA and protein decay rates (Fig. 5A′,B′), the ellipticity and asymmetry of the gradient were far more sensitive to 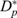 (Fig. 5C,D). This suggests that protein diffusivity has a more dominant role in establishing the medio-lateral distribution of FGF proteins than along the antero-posterior axis. This differing role of diffusion along antero-posterior and medio-lateral axes can be attributed entirely to movement of the DoT: in the limit of decreasing 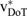 the FGF gradient becomes isotropic (*T*=*λ*=1) irrespective of 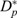 (Fig. 4D,E). Finally, although higher rates of protein production 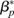 predictably increased the maximum protein concentration 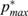, the scaled measures of antero-posterior extent and shape of the FGF gradient were otherwise insensitive to this parameter (Fig. S3).

### Chemo-mechanical coupling model reproduces key aspects of hindgut morphogenesis

Having formulated a model that describes the 2D FGF gradient in terms of transport properties of FGF transcripts and proteins, we next integrated this with a model aimed at studying the endoderm deformations that drive hindgut formation and arise in response to this gradient. Directed, collective cell movements in the posterior avian endoderm arise through conversion of the long-range FGF gradient into a force gradient via RhoA-dependent acto-myosin contractility (Nerurkar et al., 2019). By outpacing axis elongation, these endoderm movements drive the folding and elongation of the hindgut, and therefore must be appropriately coordinated with axis elongation for proper tube morphogenesis. To conceptualize this in a mathematical model, the endoderm was approximated as an active (force generating), compressible (capable of volume changes), viscous fluid with isotropic tensile stress generated in proportion to FGF concentration (obtained from the transport model). Accordingly, total stress (*σ*_*T*_) is given by the sum of a passive (*σ*_*P*_) and active (*σ*_*A*_) stress:
(5)

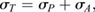
where
(6)

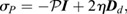
and 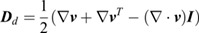 is the deviatoric component of the strain rate tensor, ***I*** is the identity tensor, ***v***=[*v*_*x*_, *v*_*y*_] is the velocity, 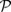 is the pressure, and *η* the viscosity. Through simultaneous measurement of single cell area and FGF activity experimentally in the endoderm, we concluded that cell area varies linearly with the inverse of FGF activity in 2D (Fig. S4). Therefore, the active stress, representing acto-myosin activity of endoderm cells generated in response to FGF (Fig. 1C), was assumed to vary linearly in proportion to FGF protein concentration:
(7)

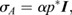
where *α* is a proportionality factor between *σ*_*A*_ and 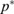 is obtained from solving Eqn 3 above. In the limit of slow flows associated with morphogenesis, we neglect inertia such that the local force balance reads:
(8)

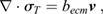
where *b*_*ecm*_ is the viscous drag coefficient resulting from interactions between endoderm cells and their basement membrane. Finally, a continuity equation relating cell contractility to volume changes during tissue flows was used to further simplify Eqns 5-8 (see supplementary Materials and Methods for further details) into:
(9)

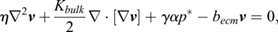
where *K*_*bulk*_ is the bulk viscosity of the endoderm and *γ* relates active in plane stress to out-of-plane motion. Upon de-dimensionalization, Eqn 9 reduces to:
(10)

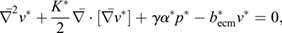
where
(11)

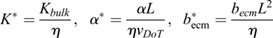
are the dimensionless parameters describing tissue mechanics, and 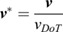.

Unlike transport parameters, which could be approximated directly from experimental measurements of FGF activity, direct quantification of mechanical properties was not feasible, and so these have been estimated from literature (Table 1).

**Fig. 6.**
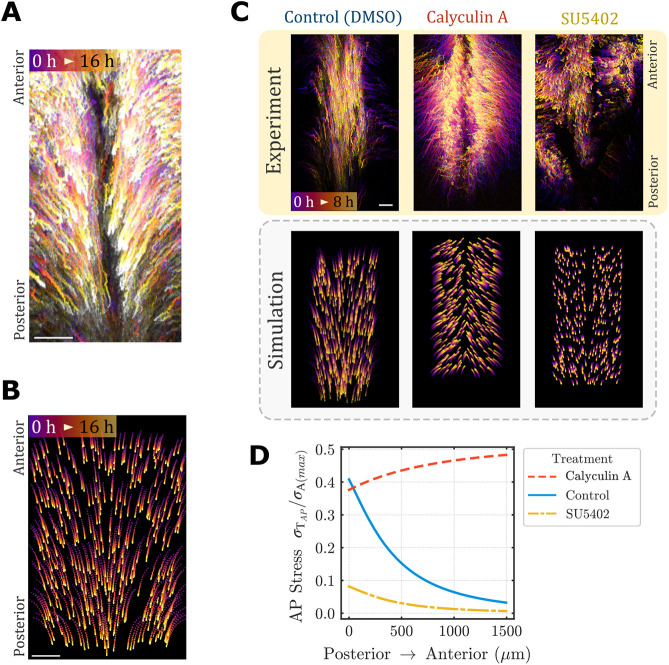
**Chemo-mechanical model replicates key aspects of avian hindgut morphogenesis.** (A) Cell tracks from *in vivo* time-lapse imaging of posterior chick endoderm cells expressing nuclear-localized GFP reporter between HH stages 13 and 18. (B) Simulated cell tracks from solution of Eqn 10. (C) Cell tracks from experiments (top) and model simulation (bottom) for pharmacological perturbations compared with DMSO control. (D) Spatial distribution of relative total anteroposterior (AP) stress *σ*_*T*_ (normalized to maximal active stress) along the embryonic midline. Scale bars: 100*μm*.

Visualizing the solution to Eqn 10 by simulating cell tracks (see supplementary Materials and Methods for further details) revealed agreement with observed cell movements in the posterior chick embryo during hindgut morphogenesis (Fig. 6A,B, Movie 2). As in the chick embryo, the model predicts endoderm movements will outpace axis elongation (Nerurkar et al., 2019). Similar to experimental observations (Nerurkar et al., 2019), the model predicted that total stresses were approximately 40% higher along the medio-lateral direction than the antero-posterior direction, and total antero-posterior stress decreases from posterior to anterior (Fig. 6D). These results suggest that the model recapitulates tissue movements and associated stresses observed *in vivo* during normal hindgut morphogenesis. For further validation, we next examined whether the model can accurately predict endoderm cell movements in response to perturbations that influence contractility or FGF activity. Treatment of the embryo with the myosin phosphatase inhibitor calyculin A remarkably reverses the direction of antero-posterior cell movements while preserving lateral-to-medial movements (Fig. 6C) (Nerurkar et al., 2019). When this was modeled as an increase in active, isotropic stress throughout the endoderm, the model successfully replicated these effects (Fig. 6C), including redirection of cells toward the anterior direction, with medial movement unaltered. In contrast to the anisotropic effects of calyculin A, experiments in which the FGF receptor inhibitor SU5402 was applied to the embryo resulted in abrogated cell movements along both medio-lateral and anteroposterior axes (Fig. 6C) (Nerurkar et al., 2019). When this perturbation was simulated as a reduction in the parameter 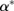, the model accurately predicted the loss of cell movements along both axes as well (Fig. 6C). Finally, the associated tissue forces also tracked with experimental findings for calyculin A and SU5402 treatments, reducing the posterior-to-anterior tension gradient, owing to an increase in anterior tension with calyculin A, and a decrease in posterior tension with SU5402 (Fig. 6D) (Nerurkar et al., 2019). In summary, the chemo-mechanical model captures essential features of normal hindgut morphogenesis, and accurately predicts the response of endoderm cell movements to experimental perturbations targeting cell contraction and FGF activity.

### Isotropic material properties drive directional cell flows in response to FGF activity gradient

We next performed parametric analyses to understand how physical properties of the endoderm influence the tissue flows organized by FGF. To simplify characterization of model simulations, three scalar metrics were considered. First, *Q*_AP_ is a bulk antero-posterior tissue flow rate providing a macroscopic readout of overall tissue movements. Second, *v*_*max*_ is the maximum (posterior-ward) speed relative to the rate of axis elongation; *v*_*max*_<1 would indicate a failure of hindgut formation due to an inability of endoderm movements to outpace axis elongation. Third, *Q*_AP_/*Q*_ML_ quantifies the anisotropy of cell movements.

**Fig. 7.**
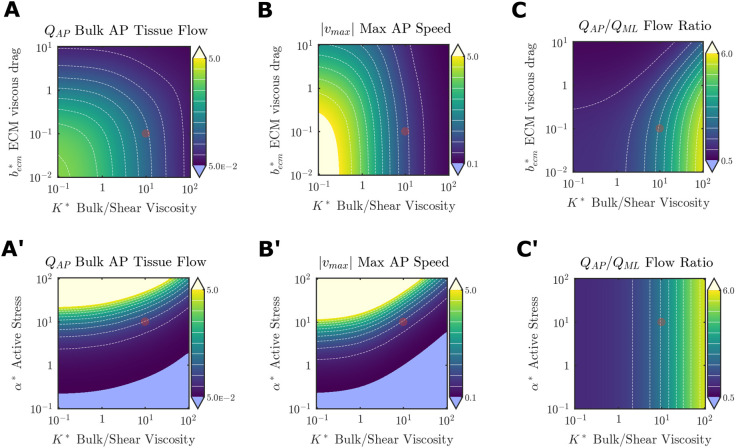
**Effects of tissue mechanics parameters on endoderm deformations.** (A-C) Sweeps of 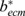 and bulk to shear viscosity ratio 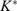. (A′-C′) Sweeps of active stress α^*^ and bulk to shear viscosity ratio K^*^. Red circle indicates baseline parameters. White dashed lines are isolines at 10% intervals of the color bar range.

We first examined how these metrics are altered when FGF transport properties are held fixed at physiological values (Table 1), and parameters describing endoderm mechanics are varied. The model considers both extrinsic (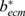) and intrinsic (K^*^) resistance to cell movements. Both had similar effects, with increases in each diminishing the influence of the other, and increasing either one reducing *Q*_AP_ and *v*_*max*_ (Fig. 7A,B). In contrast, K^*^ and 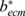 had dissimilar effects on the directionality of tissue flow: increasing K^*^ progressively biases the flow toward antero-posterior movement whereas changes in 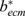 had relatively little effect (Fig. 7C). We next considered how the responsiveness of endoderm to FGF (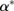) influences endoderm deformations. Bulk tissue flows and local maximum velocity increased with α^*^, confirming that increasing the conversion of FGF concentration to acto-myosin activity will coordinately produce larger deformations (Fig. 7A′,B′). Antero-posterior tissue flows were only strongly sensitive to material properties of the endoderm at high contractility (Fig. 7A′); as a more local measure of tissue movement, *v*_*max*_ was somewhat more responsive to changes in 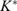 (Fig. 7B′). Anisotropy was insensitive to α^*^ (Fig. 7C′), highlighting that directionality of the movements stems from the spatial distribution of the active tension gradient, not the magnitude of active tension, which is isotropic. The effects of changing protein decay and production rates mirrored changes to α^*^ (Fig. S5).

### Axis elongation and mRNA decay strongly influence endoderm deformations driving hindgut morphogenesis

**Fig. 8.**
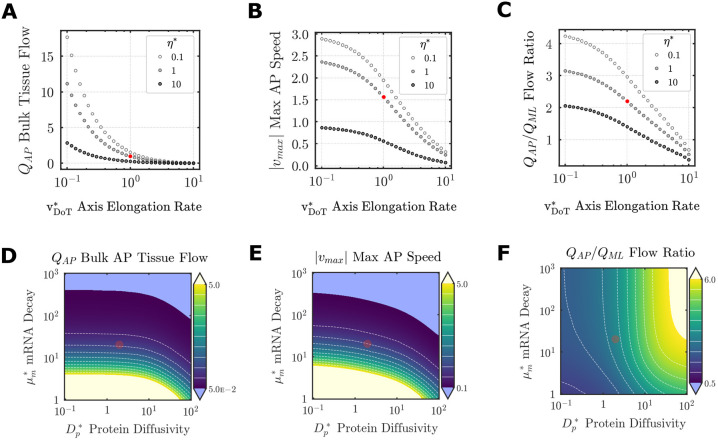
**Effects of FGF transport parameters on endoderm deformations.** (A-C) Effects of 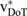 on output metrics of tissue deformation for three values of viscosity η^*^. (D-F) Effects of variation in 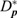 and 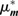 on output metrics of tissue deformation. Red circle indicates physiological/baseline parameters as measured experimentally. White dashed lines are isolines at 10% intervals of the color bar range.

Having analyzed the dependence of the chemo-mechanical model on mechanical parameters of endoderm for a given ‘physiological’ distribution of FGF ligands, we finally examined interactions between transport properties of FGF and mechanics of the endoderm. De-dimensionalization of the transport and mechanics equations led to absorption of two key properties, v_DoT_ and *η*, into the remaining model parameters. In other words, all other metrics of transport and mechanics only influence the behavior of the system via their relative magnitudes with respect to the speed of axis elongation, and endoderm shear viscosity. To examine how these two central properties interact, we performed parametric sweeps on 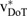 and 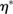 to examine the effects on tissue flows. *Q*_AP_ was highest for low 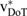 irrespective of η^*^, indicating that the largest tissue flows are achieved when the FGF gradient is nearly stationary (Fig. 8A). Increasing η^*^ reduced bulk tissue flow, yet this was largely dependent on 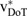; *Q*_AP_ became increasingly insensitive to changes in η^*^ as 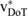 increased (Fig. 8A). Notably, the experimentally measured rate of axis elongation (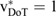) corresponds to a domain within which moderate changes in either 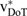 or η^*^ have relatively little influence on the total bulk flow, despite having more pronounced effects on maximum antero-posterior speed (Fig. 8B). The anisotropy of endoderm movements was strongly dependent on both 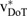 and η^*^, with each reducing anisotropy (Fig. 8C). At sufficiently high rates of axis elongation, reducing shear viscosity produced deformations in which medial flows exceed posterior ones (*Q*_AP_/*Q*_ML_<1) (Fig. 8C).

Given the importance of mRNA decay rate 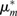 and protein diffusivity 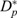 for establishment of the 2D FGF protein gradient, we next examined how variations in each influence endoderm tissue flows. Low 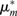 and 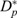, which produce an FGF gradient strongly biased along the antero-posterior axis (low *T*), also generated the largest antero-posterior flows (Fig. 8D). However, *Q*_AP_ was more sensitive to increases in 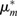 than to increases in 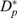, such that increases in 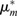 cause a pronounced decline in bulk flow and *v*_*max*_ (Fig. 8D,E). However, at sufficiently high 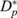, bulk tissue flow and local maximum velocity were progressively attenuated even at low 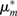. Finally, the effects of 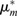 and 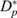 on flow anisotropy revealed a counterintuitive result: conditions that favor isotropic gradients (*T*=*λ*=1) support high anisotropy, albeit for relatively small magnitudes of tissue flow (Fig. 8D-F). This was entirely a result of the movement of the FGF gradient (in the limit of decreasing 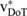 isotropic flows are recovered for isotropic FGF gradients). Together, these findings demonstrate how the mechanism of establishing a biochemical gradient is directly tied to downstream mechanics driving tissue deformations during embryogenesis.

## DISCUSSION

The present study makes use of a relatively simple 2D example of morphogen-mediated tissue deformations to develop a mathematical model that investigates the interplay between biochemical properties that inform morphogen transport and mechanical properties that relate forces to flows in the tissue. The model replicated many key experimental observations of both FGF gradient formation and endoderm cell movements (see supplementary Materials and Methods for further details), providing important qualitative validation for the approach. We found that the rate of axis elongation and transcript stability are key regulators of antero-posterior movements that drive hindgut formation, whereas protein diffusivity is limited to primarily influencing medio-lateral tissue movements. Importantly, FGF was only incorporated into the mechanics via a concentration-dependent induction of isotropic, active tension. Therefore, this simple interaction is sufficient to recreate the directional anisotropic tissue deformations that drive hindgut morphogenesis, without any other sources of anisotropy or asymmetry.

The relative shape and antero-posterior extent of the FGF protein gradient was most sensitive to the rate of axis elongation (Fig. 4B-E), and, to a somewhat lesser extent, to the decay rate of FGF transcripts (Fig. 5A-D). By contrast, the diffusion coefficient 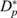, influenced the medio-lateral spread of FGF protein without a strong effect on the antero-posterior gradient (Fig. 5A′-D′). Given the importance of the antero-posterior FGF gradient for regulating many concurrent events in the posterior embryo, including somitogenesis (Dubrulle et al., 2001; Naiche et al., 2011), axis elongation (Bénazéraf et al., 2010; Regev et al., 2022; Xiong et al., 2020), Wolffian duct elongation (Atsuta and Takahashi, 2015; Attia et al., 2015) and hindgut tube formation (Nerurkar et al., 2019), these findings may offer some insight into the developmental constraints at play in the evolution of gradient formation. Protein diffusion and stability are complex properties influenced by extracellular matrix (ECM) organization and protein structure, with the latter likely constrained by the need to preserve interactions with cognate receptors across many tissues. mRNA decay, however, can be regulated with greater modularity as a cell-specific property. Indeed, there is growing evidence that transcript stability and post-transcriptional modifications may explain variations in developmental timing across species (Lázaro et al., 2023). Although morphogens have classically been assumed to form long-range gradients via diffusion from a source (Crick, 1970; Stapornwongkul and Vincent, 2021), the limited influence of protein diffusion on establishment of the antero-posterior FGF gradient is not unprecedented (Spirov et al., 2009), and advective gradient formation has been observed in other contexts as well (Ibañes et al., 2006; Pfeiffer et al., 2000). In these other contexts, as in the present study, it is likely that tissue movements that pre-pattern the mRNA distribution play a more prominent role in establishment of the protein gradient than do protein diffusion and turnover rates. Notably, diffusion has been implicated in the spread of FGF through the zebrafish gastrula (Yu et al., 2009) and in mechanically constrained zebrafish PSM explants (Simsek and Özbudak, 2018), suggesting that additional studies may be needed to reconcile those findings with the present work and prior studies in the chick PSM (Dubrulle and Pourquié, 2004) that support a limited role for diffusion.

Based on experimental measurements of FGF activity (Fig. 3), it is worth noting that the embryo occupies a neighborhood within the parameter space where the antero-posterior length of the gradient is largely insensitive to moderate changes in either mRNA or protein properties (e.g. Fig. 5B,B′), but remains sensitive to the rate of axis elongation. This may explain the highly reproducible gradient shape observed from embryo to embryo, given that presumably ‘noisier’ inputs, such as transcript and protein stability, are less influential on the extent of the gradient than is axis elongation, which is an aggregate, tissue-scale property that may be less prone to variability between individual embryos. It is also reasonable that axis elongation rate is key to gradient formation because many morphogenetic events informed by this gradient must be coordinated with axis elongation for proper establishment of the posterior body plan (Atsuta and Takahashi, 2015; Attia et al., 2015; Nerurkar et al., 2019).

Linking FGF transport to downstream tissue deformation revealed several unexpected findings, and a lack of direct one-to-one correspondence between changes in transport properties and downstream readouts of endoderm movements. For example, although ellipticity of the FGF protein gradient was sensitive to both transcript decay 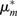 and protein diffusivity 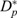 around the physiologic baseline values (e.g. Fig. 5C), the resulting effects on antero-posterior tissue flow and maximum antero-posterior velocity were largely dependent only on 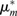. Conversely, flow anisotropy was sensitive to variation of 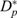 from baseline, but far less sensitive to 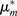.

Similar to its role in establishing the gradient, the rate of axis elongation was a key determinant of tissue deformations in the endoderm as well. For example, at sufficiently high values of 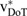, medially directed tissue flows predominated at the expense of antero-posterior movements (Fig. 8C). The interaction between 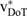 and endoderm movements is complex, owing to its influence on the system at both the mechanical and biochemical level. Increasing 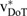 creates an elongated gradient with a lower maximum protein concentration (Fig. 4B), thereby reducing the directional bias in contractility that leads to antero-posterior movement. In addition, however, a gradient that moves too quickly relative to tissue deformations will have a diminished effect on cell movements for any given gradient shape, as cells are effectively left behind as the FGF gradient moves posteriorly. These two effects together explain the precipitous drop in antero-posterior tissue movements as 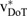 increases (Fig. 8A,B). The ability of endoderm cells to outpace axis elongation is essential for conversion of these directional cell movements into construction of a 3D epithelial tube (Nerurkar et al., 2019). Accordingly, one could interpret conditions in which *v*_*max*_<1 as unfavorable for hindgut tube formation. As expected, as 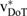 increases, ultimately cells in the endoderm are unable to keep up, suggesting that the hindgut would fail to form. Interestingly, however, this tipping point is dependent on tissue viscosity, a parameter that – on the long time scale of gut morphogenesis – reflects the bulk effects of dynamic cell–cell contacts, cell proliferation, and intrinsic viscosity of the cells themselves. At sufficiently high tissue viscosity, even extremely slow rates of axis elongation are not sufficient to drive hindgut formation (Fig. 8B). The fastest cell movements and largest antero-posterior tissue flows are achieved as 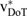 approaches zero, because this would result in the highest recruitment of cells by passive displacement of cells from low FGF concentrations to higher concentrations, where they increase acto-myosin contractility to beget even more tissue flows (Nerurkar et al., 2019). However, operation of the system well outside of this range is consistent with the idea that gut tube morphogenesis was a secondary innovation to elongation of the primary body axis by FGF signaling. Indeed, mechanisms of axis elongation are well conserved among vertebrates (McMillen and Holley, 2015; Mongera et al., 2019), but gut tube morphogenesis by distinct folding events for the foregut, hindgut and midgut is a derived trait of amniotes (Durel and Nerurkar, 2020). In other words, the posterior FGF gradient is not optimized for hindgut tube formation, but instead, hindgut morphogenesis is buffered against changes in 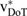, an external cue under distinct regulatory controls.

Although the model replicated key aspects of FGF gradient formation and endoderm cell movements in the chick embryo, several simplifying assumptions were made in formulation of the model to improve mathematical tractability, or because limited studies on amniote hindgut morphogenesis left little context for more complex interactions to be considered (see supplementary Materials and Methods for a detailed discussion of model assumptions, limitations, and future directions). For example, downstream activity of a promoter for the FGF target gene *Dusp6* was used as a correlate of FGF protein, owing to challenges in direct measurement of FGF ligand concentration. Further, relatively little is known regarding how upstream FGF protein abundance is potentiated to downstream acto-myosin activity. Motivated by the linear relationship between FGF activity and cell area (Fig. S4), we therefore modeled this interaction as simply as possible. It will be important to consider in future experimental work how these links are achieved, and whether intracellular signaling dynamics such as oscillatory ERK activation observed in the PSM may be involved (Simsek and Özbudak, 2018). Based on recent findings that mechanical properties are graded in the PSM (Mongera et al., 2019), it is possible that such heterogeneities exist within the endoderm as well. Although the present model does not consider such regional differences, it does demonstrate that, even in their absence, isotropic contractions are sufficient to yield anisotropic cell movements during morphogenesis of the avian hindgut.

## MATERIALS AND METHODS

### Harvesting, electroporation and *ex ovo* culture of chick embryos

Fertilized White Leghorn chicken eggs were incubated in a humidified, temperature-controlled chamber (60%, 38°C). Embryos were harvested on filter paper rings (Chapman et al., 2001) at HH stages 9 and 14, and endoderm-specific electroporation carried out as described previously (Nerurkar et al., 2019). Briefly, 5*μ*g/*μ*l of plasmid DNA in 5% sucrose and 0.1% Fast Green was injected onto the ventral surface of explanted embryos in a PBS bath, followed by electroporation with the NEPA 21 transfection system (Nepa Gene), using a pair of square electrodes with the following pulse parameters: three 40 V poring pulses of 0.1 ms duration, separated by 50 ms, with 10% decay between each pulse, followed by five 4 V transfer pulses of 5 ms duration, separated by 50 ms, with 40% decay between each pulse (Nerurkar et al., 2019).

### 2D model of FGF gradient formation

The transport model described in Eqn 3 was solved on a rectangular domain representing half of the embryo to account for bilateral symmetry of the FGF gradient (Dubrulle et al., 2001; Dubrulle and Pourquié, 2004) and endoderm cell movements (Nerurkar et al., 2019). Accordingly, the left boundary of the domain was subject to a symmetry boundary condition. The remaining three boundaries – anterior, lateral and posterior to the DoT – were assumed to be sufficiently far away that protein and mRNA concentration were zero. In addition, a finer mesh density was applied within a 2.5 mm×5 mm subdomain corresponding to the gut-forming endoderm. It was assumed that, initially, mRNA and protein concentrations are zero throughout the domain. The pair of differential equations describing mRNA and protein distributions subject to these boundary and initial conditions were solved numerically using the open-source computing platform FEniCS (Logg et al., 2012a; Kirby, 2004, 2012; Kirby and Logg, 2006; Logg and Wells, 2010; Logg et al., 2012b) to obtain time-varying spatial distributions of *Fgf8* mRNA and FGF8 protein for a given set of parameter values. The time-dependent transport equations for the protein concentration were run until a steady state solution was achieved, which was determined to have been reached after at least four successive time steps show a less than 0.5% difference from the previous time point (Fig. S1). A detailed discussion of the model methodology is provided in supplementary Materials and Methods.

### 2D model of collective movements driving hindgut formation

Conservation of mass is described using a continuity equation that accommodates area changes associated with cell contractility, relating in-plane and out-of-plane motion as (Serra et al., 2021 preprint):
(12)

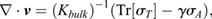
where *K*_*bulk*_ is the bulk viscosity and *γ* is a factor that controls the amount of out-of-plane motion accounted for by the active stress. Substituting Eqns 5, 6 and 7 into Eqn 12 provides an explicit relation for pressure:
(13)

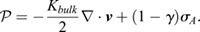
Combining Eqns 5, 8 and 13 reduces the force balance to Eqn 10 above. As with Eqn 3 describing FGF protein concentration, Eqn 10 was solved on a rectangular domain representing half of the bilaterally symmetric embryo, such that a symmetry condition was applied to the medial (left) boundary, whereas the anterior, lateral and posterior boundaries of the domain were assumed to be sufficiently distant from the region of interest that ***v***=0 was applied at each. The steady state solution of the FGF transport equations, specifically the scalar field of the protein concentration, 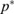, was passed to the tissue mechanics equations as an input field for defining the active stress (Eqn 7). Transport and mechanics equations were solved on the same finite element mesh in the moving frame of reference. To compare simulation results across varied input parameters, the following output metrics were defined: (1) *Q*_AP_ – bulk tissue flow in the antero-posterior axis, defined as the *y*-axis component of the velocity field integrated over the entire domain anterior to the maximal p^*^ concentration (to capture the full capacity of the solved velocity field to accommodate morphogenetic movements); (2) *v*_*max*_ – maximum posterior-ward velocity (reported as an absolute value); and (3) *Q*_AP_/*Q*_ML_ – tissue flow ratio, defined as the ratio of the antero-posterior to medio-lateral bulk tissue flow. Baseline physiologic parameter values used for parametric sweeps were derived from experimental data or estimated from the literature (Table 1), and ranges for swept parameters spanned three orders of magnitude as indicated in Table S1.

### Quantification of FGF activity gradient in the chick endoderm

Downstream activity of the FGF pathway was visualized in the posterior endoderm by electroporation of a reporter plasmid consisting of the mouse *Dusp6* promoter driving expression of either nuclear-localized eGFP or mScarlet (Ekerot et al., 2008; Nerurkar et al., 2019). The *Dusp6* reporter was co-electroporated with a ubiquitous reporter driving expression of blue fluorescent protein (BFP) to serve as an electroporation control, and FGF activity was quantified by normalizing the *Dusp6*-driven signal intensity to that of the ubiquitous reporter (Nerurkar et al., 2019; Wang et al., 2014). Electroporated embryos were imaged on an AxioZoom v1.2 macroscope (Zeiss) after 6 h of incubation. Post-processing of images was carried out using the Napari viewer (https://zenodo.org/records/7276432), Napari Assistant plugin (https://zenodo.org/records/7805849) and sci-kit image library (van der Walt et al., 2014). Briefly, Otsu's method (Otsu, 1979) was used to perform automatic image thresholding and detect *Dusp6*-driven fluorescence in cells, and maximal *Dusp6* reporter intensity values per nucleus were normalized by maximal BFP (control) intensity. The entire image was then binned into a 38×10 grid (antero-posterior×medio-lateral), and normalized *Dusp6* intensity values were averaged per bin. Estimation of the model parameters describing FGF gradient formation was carried out by curve fitting to *Dusp6* reporter data. For simplicity, we performed curve fits of an analytical solution to the 1D equivalent of Eqn 3, derived previously by Harrison and colleagues (Harrison et al., 2011) (see supplementary Materials and Methods for further details), to *Dusp6* reporter activity at HH stage 10 (*n*=9) and HH 15 (*n*=10) to obtain values for 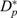, 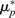 and 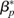 simultaneously.

To quantify FGF activity and cell shape simultaneously in the endoderm, a variant of the *Dusp6* reporter in which promoter activity drives expression of eGFP was co-electroporated with a ubiquitous reporter driving expression of BFP to serve as an electroporation control, as well as with a ubiquitous reporter driving expression of tdTomato to visualize the entire cell cytoplasm. Electroporated embryos were imaged on an AxioZoom v1.2 macroscope (Zeiss) after 6 h of incubation (HH 15, *n*=5). In addition to the ratio of maximum intensities between the *Dusp6*-eGFP and the control nuclear fluorophore (as described above), we also calculated the cell area from the tdTomato signal, whereby segmentation of cell bodies was performed manually on Napari, given the sparsity of the mosaic electroporation with three co-expressed plasmids. This allowed for straightforward correspondence of medio-lateral and antero-posterior position, FGF activity, and the cell area, on a per-cell basis (Fig. S4).

### Time-lapse imaging and simulated cell tracks

Live imaging of endoderm cell movements was performed as described previously (Nerurkar et al., 2019). In brief, embryos electroporated with pCAG-H2B-GFP, which drives nuclear-localized GFP expression under control of a ubiquitous promoter, were imaged using an inverted Zeiss LSM confocal microscope equipped with two-photon excitation at 37°C. The resulting cell tracks were visualized by depth-coded projection of the time series in Fiji. Pharmacological perturbations were performed by diluting DMSO, calyculin A and SU5402 into semisolid culture medium (Chapman et al., 2001) prior to setting, to achieve final concentrations of 0.1%, 20 nM and 50 *μ*M, respectively. To generate simulated cell tracks from the model (which as a continuum model does not consider individual cells), cells were seeded as locations in the solution field at the initial time point of the simulation, and the solved velocity field of Eqn 10 was used to compute the incremental displacement between subsequent time points, relying on interpolation via scipy.interpolate.interp2d at subsequent time points and the forward Euler method for time integration. The pharmacological treatments where simulated in the model by: (1) Calyculin A – prescribing a saturated maximum active stress along the entire definitive endoderm domain, with logistic drop-off to zero outside that domain (to avoid numerical discontinuities due to a discrete jump); and (2) SU5402 – decreasing the active contractility parameter α^*^ to a fifth of the baseline value.

### Single-cell RNA-sequencing data analysis

A previously published and publicly available data set (Ibarra-Soria et al., 2018) comprising embryonic day 8.25 mouse endoderm was analyzed to examine the spatial distribution of FGF-related genes along the antero-posterior axis (Fig. S6). Data were accessed using the online tool (marionilab.cruk.cam.ac.uk/organogenesis, accessed 2022-10-10). Analysis was carried out with the Python package Scanpy (Wolf et al., 2018), part of scverse. A diffusion-based method (scanpy.tl.dpt) was used to arrange cells in anteroposterior pseudo-space, validated by region-specific (fore-, mid-, hindgut) expression patterns.

## Supplementary Material

10.1242/develop.202010_sup1 Supplementary information

## References

[DEV202010C1] Atsuta, Y. and Takahashi, Y. (2015). FGF8 coordinates tissue elongation and cell epithelialization during early kidney tubulogenesis. *Development* 142, 2329-2337. 10.1242/dev.122408 26130757 PMC4510593

[DEV202010C2] Attia, L., Schneider, J., Yelin, R. and Schultheiss, T. M. (2015). Collective cell migration of the nephric duct requires FGF signaling. *Dev. Dyn.* 244, 157-167. 10.1002/dvdy.24241 25516335

[DEV202010C3] Baker, D. E. (2002). A geometric method for determining shape of bird eggs. *Auk* 119, 1179-1186. 10.1093/auk/119.4.1179

[DEV202010C4] Barriga, E. H., Franze, K., Charras, G. and Mayor, R. (2018). Tissue stiffening coordinates morphogenesis by triggering collective cell migration in vivo. *Nature* 554, 523-527. 10.1038/nature25742 29443958 PMC6013044

[DEV202010C5] Bénazéraf, B., Francois, P., Baker, R. E., Denans, N., Little, C. D. and Pourquié, O. (2010). A random cell motility gradient downstream of FGF controls elongation of an amniote embryo. *Nature* 466, 248-252. 10.1038/nature09151 20613841 PMC3118990

[DEV202010C6] Boulet, A. M. and Capecchi, M. R. (2012). Signaling by FGF4 and FGF8 is required for axial elongation of the mouse embryo. *Dev. Biol.* 371, 235-245. 10.1016/j.ydbio.2012.08.017 22954964 PMC3481862

[DEV202010C7] Chapman, S. C., Collignon, J., Schoenwolf, G. C. and Lumsden, A. (2001). Improved method for chick whole-embryo culture using a filter paper carrier. *Dev. Dyn.* 220, 284-289. 10.1002/1097-0177(20010301)220:3<284::AID-DVDY1102>3.0.CO;2-5 11241836

[DEV202010C8] Collinet, C. and Lecuit, T. (2021). Programmed and self-organized flow of information during morphogenesis. *Nat. Rev. Mol. Cell Biol.* 22, 245-265. 10.1038/s41580-020-00318-6 33483696

[DEV202010C9] Crick, F. (1970). Central dogma of molecular biology. *Nature* 227, 561-563. 10.1038/227561a0 4913914

[DEV202010C10] Crossley, P. H. and Martin, G. R. (1995). The mouse *Fgf8* gene encodes a family of polypeptides and is expressed in regions that direct outgrowth and patterning in the developing embryo. *Development* 121, 439-451. 10.1242/dev.121.2.439 7768185

[DEV202010C11] Desprat, N., Supatto, W., Pouille, P. A., Beaurepaire, E. and Farge, E. (2008). Tissue deformation modulates twist expression to determine anterior midgut differentiation in drosophila embryos. *Dev. Cell* 15, 470-477. 10.1016/j.devcel.2008.07.009 18804441

[DEV202010C12] Driever, W. and Nüsslein-Volhard, C. (1988). A gradient of bicoid protein in Drosophila embryos. *Cell* 54, 83-93. 10.1016/0092-8674(88)90182-1 3383244

[DEV202010C13] Dubrulle, J. and Pourquié, O. (2004). fgf8 mRNA decay establishes a gradient that couples axial elongation to patterning in the vertebrate embryo. *Nature* 427, 419-422. 10.1038/nature02216 14749824

[DEV202010C14] Dubrulle, J., McGrew, M. J. and Pourquié, O. (2001). FGF signaling controls somite boundary position and regulates segmentation clock control of spatiotemporal Hox gene activation. *Cell* 106, 219-232. 10.1016/S0092-8674(01)00437-8 11511349

[DEV202010C15] Durel, J. F. and Nerurkar, N. L. (2020). Mechanobiology of vertebrate gut morphogenesis. *Dev. Mech. Pattern. Evol.* 63, 45-52. 10.1016/j.gde.2020.04.002 PMC7483949 32413823

[DEV202010C16] Ekerot, M., Stavridis, M. P., Delavaine, L., Mitchell, M. P., Staples, C., Owens, D. M., Keenan, I. D., Dickinson, R. J., Storey, K. G. and Keyse, S. M. (2008). Negative-feedback regulation of FGF signalling by DUSP6/MKP-3 is driven by ERK1/2 and mediated by Ets factor binding to a conserved site within the DUSP6/MKP-3 gene promoter. *Biochem. J* 412, 287-298. 10.1042/BJ20071512 18321244 PMC2474557

[DEV202010C17] Forgacs, G., Foty, R. A., Shafrir, Y. and Steinberg, M. S. (1998). Viscoelastic properties of living embryonic tissues: a quantitative study. *Biophys. J.* 74, 2227-2234. 10.1016/S0006-3495(98)77932-9 9591650 PMC1299566

[DEV202010C18] Garcia, K. E., Stewart, W. G., Espinosa, M. G., Gleghorn, J. P. and Taber, L. A. (2019). Molecular and mechanical signals determine morphogenesis of the cerebral hemispheres in the chicken embryo. *Development* 146, dev174318. 10.1242/dev.174318 31604710 PMC6826035

[DEV202010C19] Godard, B. G., Dumollard, R., Munro, E., Chenevert, J., Hebras, C., McDougall, A. and Heisenberg, C.-P. (2020). Apical relaxation during mitotic rounding promotes tension-oriented cell division. *Dev. Cell* 55, 695-706.e4. 10.1016/j.devcel.2020.10.016 33207225

[DEV202010C20] Goodwin, K. and Nelson, C. M. (2021). Mechanics of development. *Dev. Cell* 56, 240-250. 10.1016/j.devcel.2020.11.025 33321105 PMC8177046

[DEV202010C21] Gordon, R., Goel, N. S., Steinberg, M. S. and Wiseman, L. L. (1972). A rheological mechanism sufficient to explain the kinetics of cell sorting. *J. Theor. Biol.* 37, 43-73. 10.1016/0022-5193(72)90114-2 4652421

[DEV202010C22] Grima, R. and Schnell, S. (2007). Can tissue surface tension drive somite formation? *Dev. Biol.* 307, 248-257. 10.1016/j.ydbio.2007.04.032 17543296 PMC1992446

[DEV202010C23] Hamburger, V. and Hamilton, H. L. (1951). A series of normal stages in the development of the chick embryo. *J. Morphol.* 88, 49-92. 10.1002/jmor.1050880104 24539719

[DEV202010C24] Harrison, N. C., del Corral, D. R. and Vasiev, B. (2011). Coordination of cell differentiation and migration in mathematical models of caudal embryonic axis extension. *PLoS One* 6, e22700. 10.1371/journal.pone.0022700 21829483 PMC3145656

[DEV202010C25] Heer, N. C., Miller, P. W., Chanet, S., Stoop, N., Dunkel, J. and Martin, A. C. (2017). Actomyosin-based tissue folding requires a multicellular myosin gradient. *Development* 144, 1876-1886. 10.1242/dev.146761 28432215 PMC5450837

[DEV202010C26] Ibañes, M., Kawakami, Y., Rasskin-Gutman, D. and Belmonte, J. C. I. (2006). Cell lineage transport: a mechanism for molecular gradient formation. *Mol. Syst. Biol.* 2, 57. 10.1038/msb4100098 17047664 PMC1682021

[DEV202010C27] Ibarra-Soria, X., Jawaid, W., Pijuan-Sala, B., Ladopoulos, V., Scialdone, A., Jörg, D. J., Tyser, R. C. V., Calero-Nieto, F. J., Mulas, C., Nichols, J., et al. (2018). Defining murine organogenesis at single-cell resolution reveals a role for the leukotriene pathway in regulating blood progenitor formation. *Nat. Cell Biol.* 20, 127-134. 10.1038/s41556-017-0013-z 29311656 PMC5787369

[DEV202010C28] Kasza, K. E., Farrell, D. L. and Zallen, J. A. (2014). Spatiotemporal control of epithelial remodeling by regulated myosin phosphorylation. *Proc. Natl. Acad. Sci. USA* 111, 11732-11737. 10.1073/pnas.1400520111 25071215 PMC4136583

[DEV202010C29] Kirby, R. C. (2004). Algorithm 839: FIAT, a new paradigm for computing finite element basis functions. *ACM Trans. Math. Softw.* 30, 502-516. 10.1145/1039813.1039820

[DEV202010C30] Kirby, R. C. (2012). FIAT: numerical construction of finite element basis functions. In *Automated Solution of Differential Equations by the Finite Element Method, volume 84 of Lecture Notes in Computational Science and Engineering* (ed. K.-A. M. A. Logg and G. N. Wells). Section: 13. Springer.

[DEV202010C31] Kirby, R. C. and Logg, A. (2006). A compiler for variational forms. *ACM Trans. Math. Softw.* 32, 417-444. 10.1145/1163641.1163644

[DEV202010C32] Lázaro, J., Costanzo, M., Sanaki-Matsumiya, M., Girardot, C., Hayashi, M., Hayashi, K., Diecke, S., Hildebrandt, T. B., Lazzari, G., Wu, J. et al. (2023). A stem cell zoo uncovers intracellular scaling of developmental tempo across mammals. *Cell Stem Cell* 30, 938-949.e7. 10.1016/j.stem.2023.05.014 37343565 PMC10321541

[DEV202010C33] Lecuit, T. and Yap, A. S. (2015). E-cadherin junctions as active mechanical integrators in tissue dynamics. *Nat. Cell Biol.* 17, 533-539. 10.1038/ncb3136 25925582

[DEV202010C34] Logg, A. and Wells, G. N. (2010). DOLFIN: automated finite element computing. *ACM Trans. Math. Softw.* 37, 1-28. 10.1145/1731022.1731030

[DEV202010C35] Logg, A., Wells, G. and Mardal, K.-A. (2012a). *Automated Solution of Differential Equations by the Finite Element Method*. Springer.

[DEV202010C36] Logg, A., Wells, G. N., Rognes, M. E. and Ølgaard, K. B. (2012b). FFC: the FEniCS Form Compiler. In *Automated Solution of Differential Equations by the Finite Element Method, volume 84 of Lecture Notes in Computational Science and Engineering* (ed. K.-A. M. A. Logg and G. N. Wells). Section: 11, Springer.

[DEV202010C37] McMillen, P. and Holley, S. A. (2015). The tissue mechanics of vertebrate body elongation and segmentation. *Curr. Opin. Genet. Dev.* 32, 106-111. 10.1016/j.gde.2015.02.00 25796079 PMC4470730

[DEV202010C38] Mongera, A., Michaut, A., Guillot, C., Xiong, F. and Pourquié, O. (2019). Mechanics of anteroposterior axis formation in vertebrates. *Annu. Rev. Cell Dev. Biol.* 35, 259-283. 10.1146/annurev-cellbio-100818-125436 31412208 PMC7394480

[DEV202010C39] Müller, P., Rogers, K. W., Jordan, B. M., Lee, J. S., Robson, D., Ramanathan, S. and Schier, A. F. (2012). Differential diffusivity of nodal and lefty underlies a reaction-diffusion patterning system. *Science* 336, 721-724. 10.1126/science.1221920 22499809 PMC3525670

[DEV202010C40] Naiche, L. A., Holder, N. and Lewandoski, M. (2011). FGF4 and FGF8 comprise the wavefront activity that controls somitogenesis. *Proc. Natl. Acad. Sci. USA* 108, 4018-4023. 10.1073/pnas.1007417108 21368122 PMC3054031

[DEV202010C41] Nelson, C. M. and Gleghorn, J. P. (2012). Sculpting organs: mechanical regulation of tissue development. *Annu. Rev. Biomed. Eng.* 14, 129-154. 10.1146/annurev-bioeng-071811-150043 22524386

[DEV202010C42] Nerurkar, N. L., Lee, C. H., Mahadevan, L. and Tabin, C. J. (2019). Molecular control of macroscopic forces drives formation of the vertebrate hindgut. *Nature* 565, 480-484. 10.1038/s41586-018-0865-9 30651642 PMC6397660

[DEV202010C43] Otsu, N. (1979). A threshold selection method from gray-level histograms. *IEEE Trans. Syst. Man Cybern.* 9, 62-66. 10.1109/TSMC.1979.4310076

[DEV202010C44] Pan, Y., Heemskerk, I., Ibar, C., Shraiman, B. I. and Irvine, K. D. (2016). Differential growth triggers mechanical feedback that elevates Hippo signaling. *Proc. Natl. Acad. Sci. USA* 113, E6974-E6983. 10.1073/pnas.1615012113 27791172 PMC5111668

[DEV202010C45] Pfeiffer, S., Alexandre, C., Calleja, M. and Vincent, J.-P. (2000). The progeny of wingless-expressing cells deliver the signal at a distance in Drosophila embryos. *Curr. Biol.* 10, 321-324. 10.1016/S0960-9822(00)00381-X 10744976

[DEV202010C46] Regev, I., Guevorkian, K., Gupta, A., Pourquié, O. and Mahadevan, L. (2022). Rectified random cell motility as a mechanism for embryo elongation. *Development* 149, dev199423. 10.1242/dev.199423 35344041 PMC9017234

[DEV202010C47] Saez, A., Anon, E., Ghibaudo, M., Du Roure, O., Di Meglio, J.-M., Hersen, P., Silberzan, P., Buguin, A. and Ladoux, B. (2010). Traction forces exerted by epithelial cell sheets. *"J. Phys., Condens. Matter."* 22, 194119. 10.1088/0953-8984/22/19/194119 21386442

[DEV202010C48] Savin, T., Kurpios, N. A., Shyer, A. E., Florescu, P., Liang, H., Mahadevan, L. and Tabin, C. J. (2011). On the growth and form of the gut. *Nature* 476, 57-62. 10.1038/nature10277 21814276 PMC3335276

[DEV202010C49] Sawada, A., Shinya, M., Jiang, Y.-J., Kawakami, A., Kuroiwa, A. and Takeda, H. (2001). Fgf/MAPK signalling is a crucial positional cue in somite boundary formation. *Development* 128, 4873-4880. 10.1242/dev.128.23.4873 11731466

[DEV202010C50] Serra, M., Nájera, G. S., Chuai, M., Spandan, V., Weijer, C. J. and Mahadevan, L. (2021). A mechanochemical model recapitulates distinct vertebrate gastrulation modes. *bioRxiv. 2021.10.03.462928*. 10.1101/2021.10.03.462928 PMC10699781 38055823

[DEV202010C51] Shyer, A. E., Tallinen, T., Nerurkar, N. L., Wei, Z., Gil, E. S., Kaplan, D. L., Tabin, C. J. and Mahadevan, L. (2013). Villification: how the gut gets its villi. *Science* 342, 212-218. 10.1126/science.1238842 23989955 PMC4045245

[DEV202010C52] Simsek, M. F. and Özbudak, E. M. (2018). Spatial fold change of FGF signaling encodes positional information for segmental determination in zebrafish. *Cell Rep.* 24, 66-78.e8. 10.1016/j.celrep.2018.06.023 29972792 PMC6063364

[DEV202010C53] Spirov, A., Fahmy, K., Schneider, M., Frei, E., Noll, M. and Baumgartner, S. (2009). Formation of the *bicoid* morphogen gradient: an mRNA gradient dictates the protein gradient. *Development* 136, 605-614. 10.1242/dev.031195 19168676 PMC2685955

[DEV202010C54] Stapornwongkul, K. S. and Vincent, J.-P. (2021). Generation of extracellular morphogen gradients: the case for diffusion. *Nat. Rev. Genet.* 22, 393-411. 10.1038/s41576-021-00342-y 33767424

[DEV202010C55] Valet, M., Siggia, E. D. and Brivanlou, A. H. (2022). Mechanical regulation of early vertebrate embryogenesis. *Nat. Rev. Mol. Cell Biol.* 23, 169-184. 10.1038/s41580-021-00424-z 34754086

[DEV202010C56] van der Walt, S., Schönberger, J. L., Nunez-Iglesias, J., Boulogne, F., Warner, J. D., Yager, N., Gouillart, E. and Yu, T. (2014). scikit-image: image processing in Python. *PeerJ* 2, e453. 10.7717/peerj.453 25024921 PMC4081273

[DEV202010C57] Walton, K. D., Whidden, M., Kolterud, A., Shoffner, S., Czerwinski, M. J., Kushwaha, J., Parmar, N., Chandhrasekhar, D., Freddo, A. M., Schnell, S., et al. (2015). Villification in the mouse: Bmp signals control intestinal villus patterning. *Development* 143, 734-764. 10.1242/dev.130112 PMC4760312 26721501

[DEV202010C58] Wang, S., Sengel, C., Emerson, M. and Cepko, C. (2014). A gene regulatory network controls the binary fate decision of rod and bipolar cells in the vertebrate retina. *Dev. Cell* 30, 513-527. 10.1016/j.devcel.2014.07.018 25155555 PMC4304698

[DEV202010C59] Wolf, F. A., Angerer, P. and Theis, F. J. (2018). SCANPY: large-scale single-cell gene expression data analysis. *Genome Biol.* 19, 15. 10.1186/s13059-017-1382-0 29409532 PMC5802054

[DEV202010C60] Wolpert, L. (1969). Positional information and the spatial pattern of cellular differentiation. *J. Theor. Biol.* 25, 1-47. 10.1016/S0022-5193(69)80016-0 4390734

[DEV202010C61] Xiong, F., Ma, W., Bénazéraf, B., Mahadevan, L. and Pourquié, O. (2020). Mechanical coupling coordinates the co-elongation of axial and paraxial tissues in avian embryos. *Dev. Cell* 55, 354-366.e5. 10.1016/j.devcel.2020.08.007 32918876 PMC7685225

[DEV202010C62] Yu, S. R., Burkhardt, M., Nowak, M., Ries, J., Petrásek, Z., Scholpp, S., Schwille, P. and Brand, M. (2009). Fgf8 morphogen gradient forms by a source-sink mechanism with freely diffusing molecules. *Nature* 461, 533-536. 10.1038/nature08391 19741606

[DEV202010C63] Zorn, A. M. and Wells, J. M. (2009). Vertebrate endoderm development and organ formation. *Annu. Rev. Cell Dev. Biol.* 25, 221-251. 10.1146/annurev.cellbio.042308.113344 19575677 PMC2861293

