# Supplementary material for: A chemo-mechanical model of endoderm movements driving elongation of the amniote hindgut
Source: Development. 2023 Nov 16;150(22):dev202010. doi: 10.1242/dev.202010 (PMC10690059; doi:10.1242/dev.202010)
Supplement: Supplementary information [file develop-150-202010-s1.pdf]

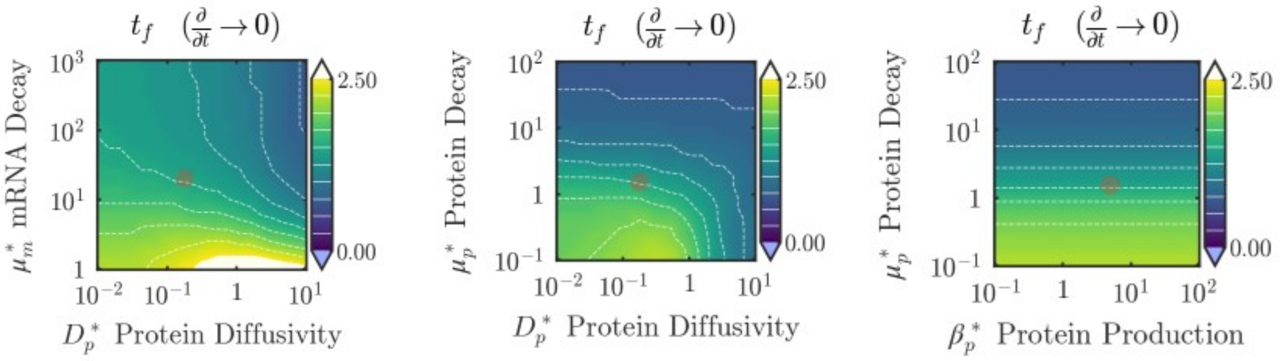

Fig. S1. Time to steady state for the swept range of the transport parameters (all simulations on FGF transport equations were run until steady state was reached; step changes in isolines are the result of coarse time steppping, as primary focus was on steady state behavior. Red circle indicates physiologic/baseline parameter values.

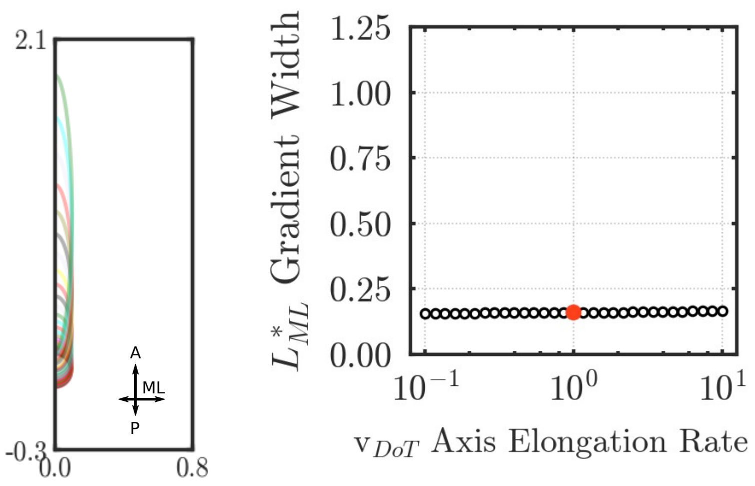

Fig. S2. (**A**) Parametric sweep of v^*_{DoT} and effects on FGF gradient shape (complementary to Fig 4). Isolines indicating $p^* = p^*_{max}/e$ for a range of v^*_{DoT} values. Isolines are pseudocolored for visibility. (**B**) Normalized medio-lateral extent L^*_{ML} of the FGF gradient for a range of v^* values.

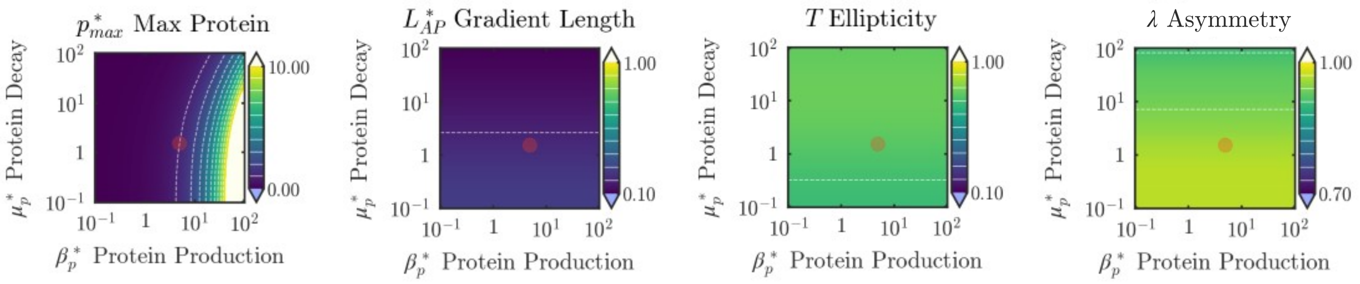

Fig. S3. Effects of variation in protein production β^*_p and protein decay rate μ^*_p on the FGF gradient. Red circle indicates physiologic/baseline parameter values.

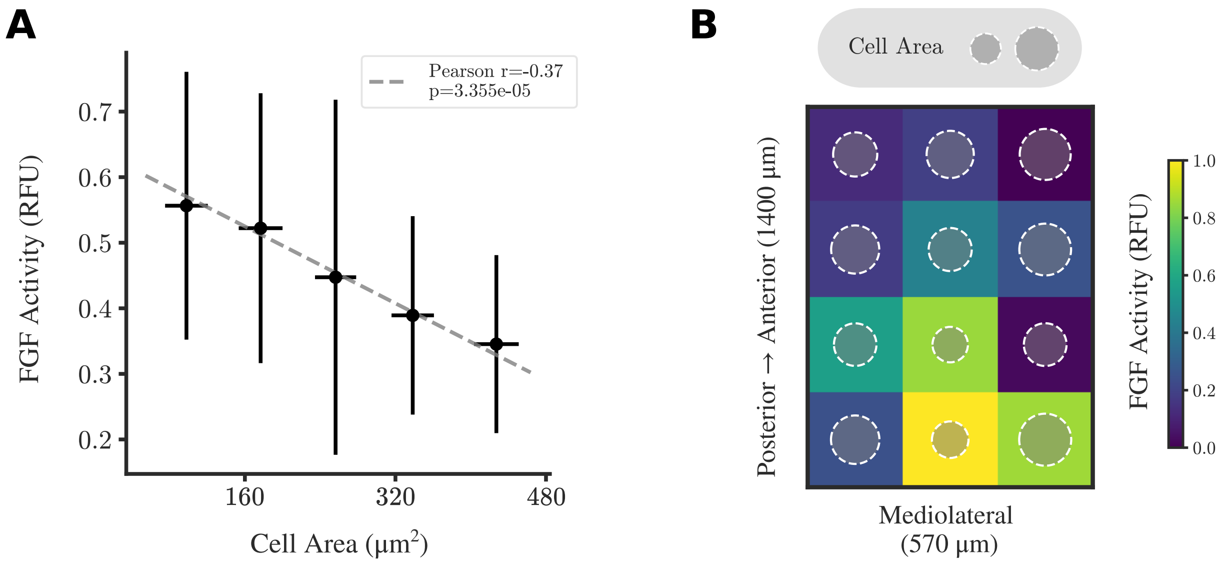

Fig. S4. FGF activity is inversely correlated with cell area in 2-D (n = 5 embryos). (**A**) Pooling of cells across the medio-lateral and antero-posterior extent of the posterior definitive endoderm reveals an inverse correlation of FGF Activity (via Dusp6 reporter) and cell area. (**B**) 2-D heatmaps of paired FGF Activity and cell shape measurements reported in (A), where binning reveals both medio-lateral and posterior-to-anterior gradients in FGF activity that are inversely related to cell area; the central column coincides with embryonic midline. No statistical method was used to determine sample size a priori.

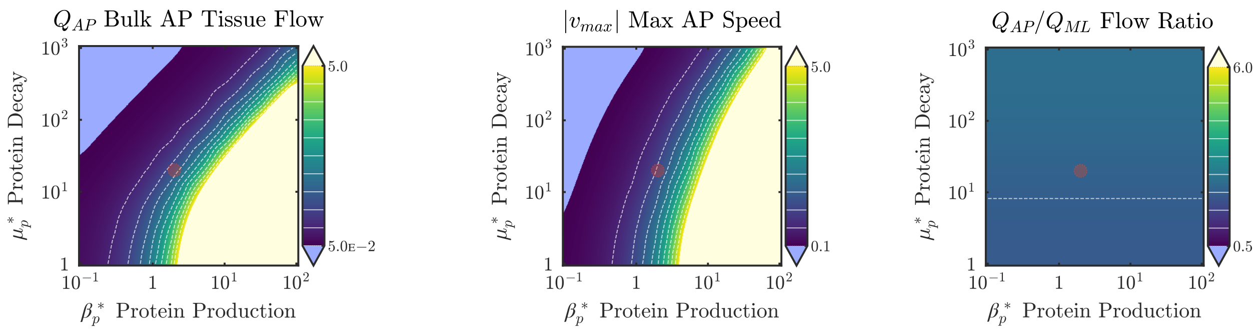

Fig. S5. Effects of variation in protein production $\beta_p{}^*$ and protein decay $\mu^*{}_p$ on the tissue movements. Red circle indicates physiologic/baseline parameter values.

Development • Supplementary information

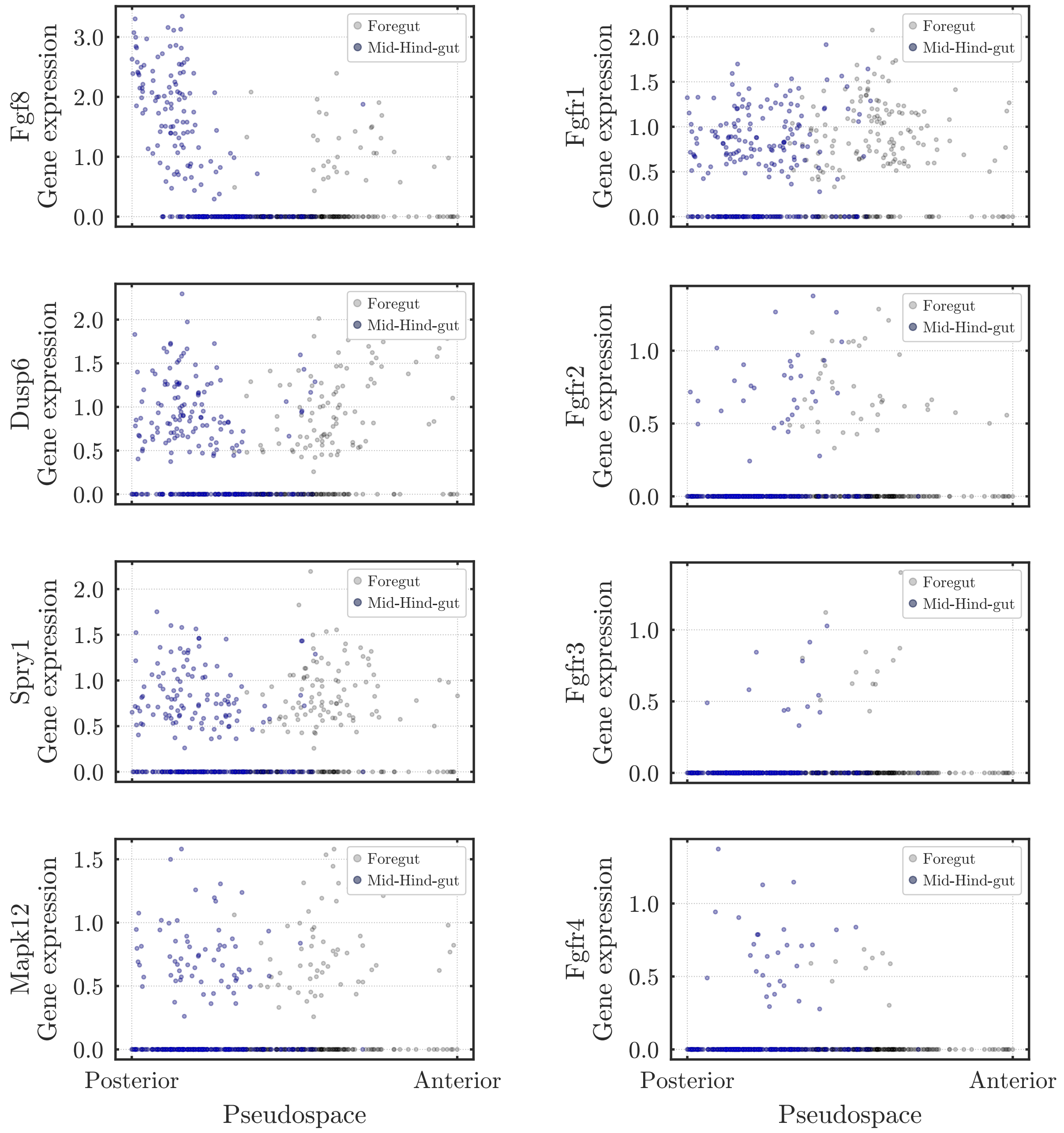

Fig. S6. Gene expression trends (reported in log(TranscriptsPerMillion) units) per cell plotted along inferred antero-posterior axis (cells arranged in pseudo-space). Left column: FGF ligand (FGF8) and donwstream effectors (Dusp6, Spry1, Mapk12) of the pathway; right column: FGF receptors. Dataset comes from E8.25 mouse endoderm (Ibarra-Soria et al., 2018).

Table S1. Baseline values used for dimensionless parameters in simulations (and estimated experimentally for $D^*_p, \mu^*_p, \beta^*_p$). v^*_{DoT} and η^* represent scaled parameters normalized to experimentally measured axis elongation rate and an arbitrary reference viscosity, respectively. †Ranges (for parametric sweeps) are reported in log-10-space as [start, stop, steps].

Parameter	Value	Range†
**FGF Transport**		
D^*_p	0.18	[-1,2,25]
μ^*_p	1.5	[-1,2,25]
β^*_p	5	[-1,2,25]
μ^*_m	20	[0,3,25]
v^*_{DoT}	1	[-1,1,31]
**Tissue Mechanics**		
η^*	1	[-1,2,25]
K^*	10	[-1,2,25]
b^*_{ecm}	0.1	[-2,1,25]
α^*	10	[-1,2,25]

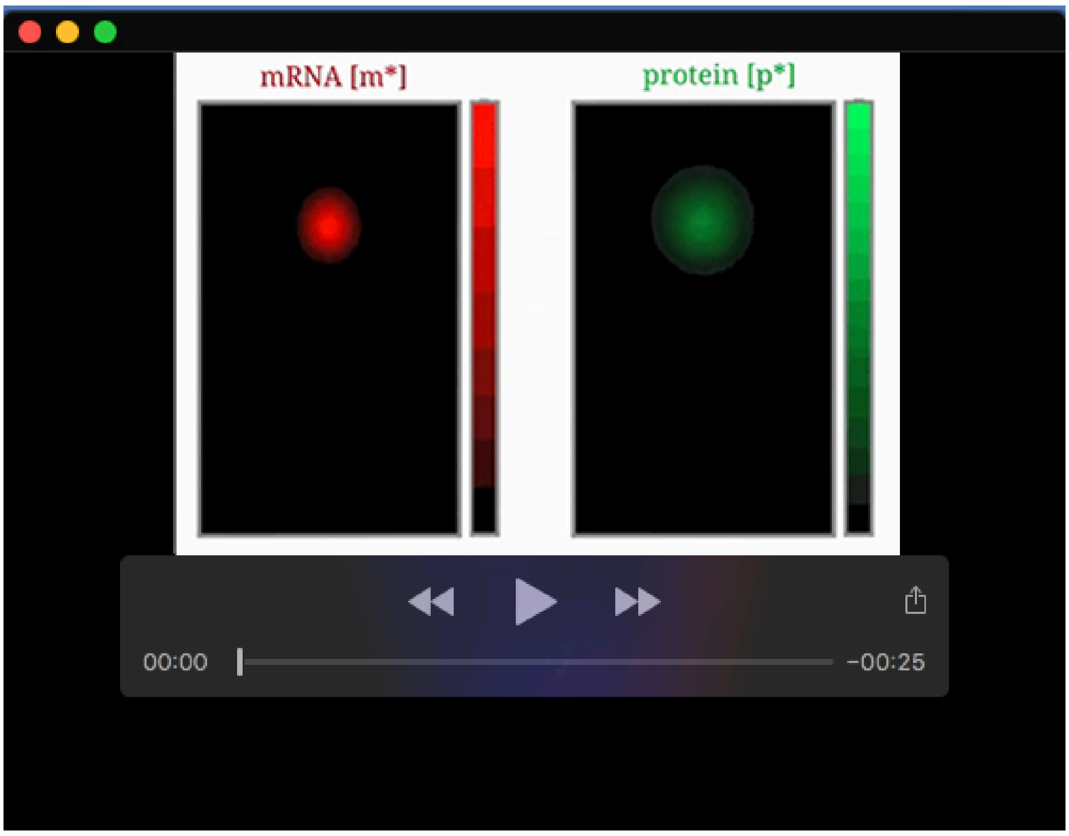

Movie 1. Simulation of mRNA and protein concentration dynamics (in the laboratory frame of reference).

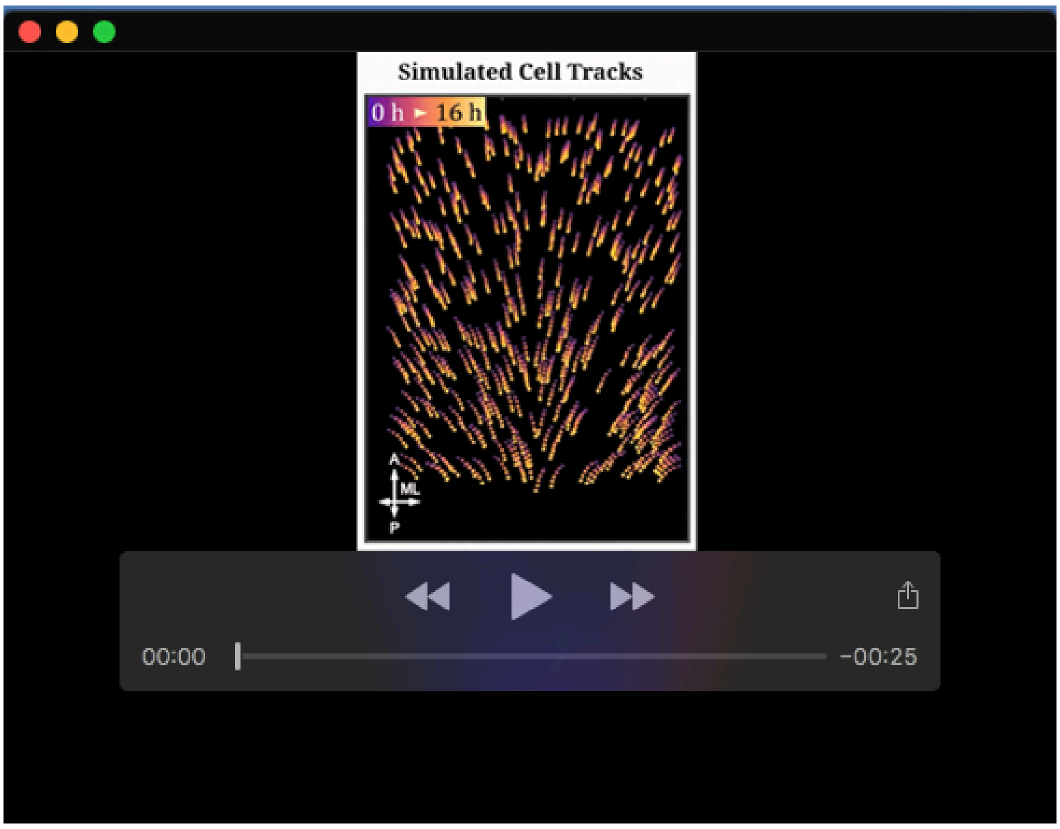

Movie 2. Simulated cell tracks with temporal color coding.

Supplementary Materials and Methods

Mathematical modeling overview

The present study makes use of a mathematical model to study how transport properties of a morphogen are integrated with downstream tissue mechanics to coordinate morphogenesis, focusing on collective cell movements in the avian endoderm. These movements result from conversion of a biochemical gradient of FGF proteins into an active force gradient via acto-myosin based cell shape changes (Nerurkar et al., 2019). Our modeling approach relies on coupling partial differential equations of transport phenomena describing the establishment and maintenance of an FGF gradient together with continuum mechanics equations describing flows of the tissue that result from active force generation. We first formulate balance equations that equate the rates of production, destruction, and movement for each mRNA and protein with their rates of accumulation or depletion. Solving these equations provides mRNA and protein concentrations as a function of time and position in two dimensions. The protein concentration is then fed into a second set of equations describing tissue mechanics, where tensile stress is generated in proportion to protein concentration and the effects on tissue flows are analyzed. The derivation and solution of these coupled equations is described in detail in the subsections below.

The present model has been shaped by our prior experimental studies and a simple 1-D mathematical model (Nerurkar et al., 2019). While the 1-D model did highlight key aspects of the system, it was limited in a number of ways, and addressing those limitations has guided the modeling approach we take in the present work. For example, prior experiments suggested that isotropic contraction is sufficient to generate anisotropic cell movements when contractility is spatially graded, but this could not be investigated in a 1-D model. Therefore, to test this directly, the present modeling approach considers 2-D tissue deformations and FGF gradient anisotropy. Our that the gradient itself moves posteriorly during axis elongation. To study how hindgut formation is coordinated with axis elongation, and to ask whether physical properties of the endoderm are buffered against or optimized for external constraints imposed by the dynamics of axis elongation, the present approach captures movement of the gradient as separate from that of the endoderm. Accounting for axis elongation is also necessary to define the FGF gradient in terms of biophysical parameters such as diffusion, transcript and protein degradation, and translation rates; a balance law that relied on these parameters but did not account for movement of the zone of active transcription would fail to replicate the mechanism of gradient formation or its final shape. Defining the FGF gradient in this way, rather than the more empirical approach of lumping these properties into a generic exponential decay function (Regev et al., 2022) is necessary to test how specific transport properties of FGF can influence cell movements and their dependence on mechanical properties. The present approach also makes important improvements to the material properties of the endoderm and its extracellular matrix. Owing to the relatively large deformations and gradients in shear strain observed during hindgut formation (Nerurkar et al., 2019), we describe the endoderm here as an active fluid, rather than a viscoelastic solid. This is consistent with the idea that on the long time scales of morphogenesis, the tissue reorganizes and deforms to dissipate stored energy, which we have observed experimentally as a limited recoil upon disruption of contractility (Nerurkar et al., 2019). Similarly, the extracellular matrix is treated as a viscous drag rather than a spring, recognizing that it is unlikely that the large deformations observed would be shared by an elastically deforming basement membrane without considerable dissipation of stored energy.

Previous one-dimensional mathematical models of FGF gradient formation have provided insights into the underlying mechanisms that balance the persistence of a transcriptionally active progenitor domain with axis elongation (N. C. Harrison et al., 2011) and dynamic consequences of the protein gradient for somitogenesis (R. E. Baker et al., 2008). However, because the FGF gradient has largely been studied in relation to somitogenesis and axis-elongation, where medio-lateral extent of the gradient may not play a major role, the focus has primarily been on the 1-D, antero-posterior features of the gradient. As a result, less attention has been paid to the mechanisms of establishing 2-D gradient anisotropy. However, in the hindgut, where this anisotropy is central to establishing directional endoderm cell movements, it is necessary to consider how two-dimensional aspects of the FGF gradient inform the mechanics of cell movement.

FGF transport – mRNA decay model

The 2-D mRNA decay model developed in the present study is of the reaction-diffusion type. In the most general form, we can write the following balance equation for the time dependent behavior of the concentration u

Development • Supplementary information

for a given chemical species of interest:

$$\frac{\partial u}{\partial t} = \underbrace{\nabla \cdot (\mathbf{D}\nabla u)}_{\text{Diffusion}} + \underbrace{\nabla \cdot (u\mathbf{v})}_{\text{Advection}} + \underbrace{f(u)}_{\text{Reaction}}$$

This general form of partial differential equation was used to model two species, *Fgf* mRNA (m) and the protein (p), defining the appropriate reaction term for each species. Previous studies show that *FGF8* transcription is not altered by FGF signaling, and hence there is no need for an autocatalytic term in the equations (N. C. Harrison et al., 2011). We assume that mRNA does not diffuse, and that protein diffuses isotropically (i.e. $\mathbf{D_p} = D_p\mathbf{I}$ which simplifies the diffusion term to: $\nabla \cdot (D_p \nabla p) = D_p \nabla^2 p$. Because the model is derived and solved in a frame of reference that moves with the domain of active transcription, an advection term is introduced to account for mass flux relative to the frame of reference. The velocity of the tailbud is approximated as constant and one-dimensional (Xiong et al., 2020), namely $\mathbf{v} = [v_x, v_y] = [0, \mathrm{v}_{\mathrm{DoT}}]$, and so the advection terms for both species simplifies to $\nabla \cdot (u\mathrm{v}_{\mathrm{DoT}}) = \mathrm{v}_{\mathrm{DoT}} \frac{\partial u}{\partial y}$. Lastly, the mRNA has a constant production rate r_m, which is modelled as a constant 2-D Gaussian, whereas the production of the protein depends on the mRNA levels as $\beta_p m$, and both mRNA and protein decay in proportion to their respective concentrations, with decay rates of μ_m, μ_p. Combining these terms into the generalized diffusion-advection-reaction equations above results in the following pair of coupled partial differential equations:

$$\begin{aligned} \frac{\partial m}{\partial t} &= \mathrm{v}_{\mathrm{DoT}} \frac{\partial m}{\partial y} + r_m - \mu_m m \\ \frac{\partial p}{\partial t} &= D_p \nabla^2 p + \mathrm{v}_{\mathrm{DoT}} \frac{\partial p}{\partial y} + \beta_p m - \mu_p p \end{aligned} \tag{S1}$$

De-dimensionalization, boundary conditions, and solution domain

We next de-dimensionalized these equations to distill them to their simplest form. Recognizing the dimension of variables and constants as:

$$\begin{aligned} [p], [m] &\sim L^{-3} \\ [r_m] &\sim L^{-3}T^{-1} \\ [\beta_p], [\mu_m], [\mu_p] &\sim T^{-1} \\ [D_p] &\sim L^2T^{-1} \\ [\mathrm{v}_{\mathrm{DoT}}] &\sim LT^{-1} \end{aligned} \tag{S2}$$

We define scaled variables:

$$x^* = \frac{x}{L}\ , \quad y^* = \frac{y}{L}\ , \quad t^* = \frac{t}{\tau} = \frac{\mathrm{v}_{\mathrm{DoT}}}{L} t \tag{S3}$$

where L and τ are the characteristic length and time scale, respectively.

Eq. S1 can then be written in non-dimensional form as:

$$\begin{aligned} \frac{\partial m^*}{\partial t} &= \frac{\partial m^*}{\partial y} + r_m^* - \mu_m m^* \\ \frac{\partial p^*}{\partial t} &= D_p^* \nabla^2 p^* + \frac{\partial p^*}{\partial y} + \beta_p^* m^* - \mu_p^* p^* \end{aligned} \tag{S4}$$

where * denotes a dimensionless variable or parameter. The de-dimensionalization procedure yielded the following non-dimensional parameters:

$$D_p^* = \frac{D_p}{\mathrm{v}_{\mathrm{DoT}} L}, \quad \beta_p^* = \frac{\beta_p L}{\mathrm{v}_{\mathrm{DoT}}}, \quad \mu_p^* = \frac{\mu_p L}{\mathrm{v}_{\mathrm{DoT}}}, \quad \mu_m^* = \frac{\mu_m L}{\mathrm{v}_{\mathrm{DoT}}}$$

Development • Supplementary information

The associated boundary and initial conditions for Eq. S1 are:

$$\begin{aligned}
&\textbf{BCs:} && m^*\Big|_{\partial\Omega} = p^*\Big|_{\partial\Omega} = 0 \\
&\text{except} && \frac{\partial m^*}{\partial x}\Big|_{x=0} = \frac{\partial p^*}{\partial x}\Big|_{x=0} = 0 \ \ \text{(for L-R symmetry boundary)} \\
&\textbf{ICs:} && m^*\Big|_{t=0} = p^*\Big|_{t=0} = 0
\end{aligned} \tag{S5}$$

where domain $\Omega = [0, 2L] \times [-2L, 10L]$ (antero-posterior $\times$ medio-lateral) and $\partial\Omega$ is the domain boundary. Note, we also define a subdomain of Ω, $\Omega_{em} = [0, 0.25L] \times [0, L]$, which corresponds to the area occupied by the embryo proper (as opposed to extra-embryonic tissue, e.g. hypoblast), and is where the steady-state criterion for the solution is evaluated. Size of the domain Ω was chosen to be more than an order of magnitude larger than the effective range of the FGF gradient in order to minimize the influence of artificially constraining boundary conditions on the solution. As a result, the solution is somewhat insulated from the requirement that mRNA and protein concentrations vanish on the anterior, lateral, and posterior boundaries; it is also consistent with experimental observations that suggest FGF ligand expression and downstream pathway activity are each extinguished well within the imposed boundary of our model. Finally, because the system of equations was formulated in a frame of reference that moves with the DoT, and in a domain that is much larger than the region of interest, it was possible to capture the full dynamics of gradient formation (Fig. 2B) without the need for a domain that grows as the embryo elongates.

Baseline/physiologic parameter values

Physiologic values of the non-dimensional parameters above were estimated by fitting the model to experimental data for FGF activity, quantified from electroporation of the endoderm with the Dusp6 reporter (dashed lines, Fig. 3B). Specifically, to fit the protein kinetic parameters (D_p, μ_p, β_p) to our experimental data (using the `scipy.optimize.curve fit`_function), the analytical solution of the 1-D reaction-diffusion-advection problem from (N. C. Harrison et al., 2011) was used:

$$p = \begin{cases}
\frac{\beta_p}{D_p(\lambda_1-\lambda_2)} \left\{ \left(\frac{\lambda_2-\lambda_1}{(\xi-\lambda_2)(\xi-\lambda_1)} \right) e^{\xi x} + \left(\frac{1}{\xi-\lambda_1} + \frac{\left(1-e^{-\lambda_1 a}\right)}{\lambda_1} \right) e^{\lambda_1 x} \right\} & \text{for} \quad x \le 0 \\
\frac{\beta_p}{D_p(\lambda_1-\lambda_2)} \left\{ \frac{e^{\lambda_2 x}}{\xi-\lambda_2} + \frac{e_2\lambda_2-1}{\lambda_2} + \frac{1-e^{\lambda_1(x-a)}}{\lambda_1} \right\} & \text{for} \quad 0 \le x \le a \\
\frac{\beta_p}{D_p(\lambda_1-\lambda_2)} \left(\frac{1}{\xi-\lambda_2} + \frac{1-e^{-\lambda_2 a}}{\lambda_2} \right) e^{\lambda_2 x} & \text{for} \quad x > a
\end{cases}$$

where $\lambda_{1,2} = -\frac{v_{DoT}}{2D_p} \pm \frac{\sqrt{v_{DoT}^2+4\mu_p D_p}}{2D_p}$, a = size of DoT, and $\xi = \frac{\mu_m}{v_{DoT}}$

In the above equation, p corresponds to the protein concentration, and $v_{DoT}, \mu_m, D_p, \beta_p, \mu_p$ are defined as above; detailed solution of the 1-D problem can be found in the original publication (N. C. Harrison et al., 2011). Best-fit values of each parameter were insensitive to initialization values spanning 4 orders of magnitudes, confirming that curvefits reflected global rather than local minima. To complete the fitting procedure, we used an initial guess of the mRNA decay parameter μ_m, which was then revised via 2-D correlation to experimental results (Fig. 3D, E).

To extract shape descriptors of the FGF gradient, an isoline at $1/e$ of the maximal concentration was fit to the ovoid function (D. E. Baker, 2002), using the `scipy.optimize.curve fit` function. The ovoid function offers a simple formula to approximate asymmetric elliptical shapes (similar to those observed experimentally), with the ellipticity T encoding the shape aspect ratio, and the asymmetry λ the reciprocal blunting of one end and sharpening of the opposing end of the shape.

Development • Supplementary information

Tissue Mechanics – Active fluid model

To model endoderm mechanics, we use the theory of active fluids – the epithelium is modeled as a viscous fluid, with active tensile stress modulated by the concentration of FGF protein obtained from solving the FGF transport model described above.

On the long time scales over which morphogenesis occurs, inertial effects can be ignored so that local force balance reads

$$\nabla \cdot \sigma_T = b_{ecm}\mathbf{v}$$

where the total stress $\sigma_T = \sigma_P + \sigma_A$ is the sum of the passive stress and the active stress.
The active stress is fully isotropic and reads:

$$\sigma_A = \alpha p^* \mathbf{I}$$

where p^* is the FGF concentration as computed by the FGF transport model above and α is a factor to convert from units of concentration to units of stress.
The passive stress can be further decomposed as:

$$\sigma_P = -\mathcal{P}\mathbf{I} + 2\eta \mathbf{D}_d$$

where $\mathbf{D}_d = \frac{1}{2}\left(\nabla\mathbf{v} + \nabla\mathbf{v}^T - (\nabla\cdot\mathbf{v})\mathbf{I}\right)$; $\mathbf{v} = [v_x, v_y]$ is the velocity, $\mathcal{P}$ is the pressure and η the shear viscosity.

We model the fluid as compressible, to permit area changes in 2-D that are accommodated by out of plane movements (i.e. to allow negative in-plane divergence) ((Serra et al., 2021); preprint):

$$\begin{aligned}
\nabla\cdot\mathbf{v} &= (K_{bulk})^{-1}(\mathrm{Tr}[\sigma_T] - \gamma\sigma_A) \\
\nabla\cdot\mathbf{v} &= 2(K_{bulk})^{-1}(-\mathcal{P} + \sigma_\alpha - \gamma\sigma_A) \\
\implies \mathcal{P} &= -\frac{K_{bulk}}{2}\nabla\cdot\mathbf{v} + (1-\gamma)\sigma_A
\end{aligned} \tag{S6}$$

where K_{bulk} is the bulk viscosity and γ a factor that controls the amount of out of plane motion due to active stress. Unlike shear viscosity η, which quantifies the resistance of a fluid to shear forces associated with friction between fluid 'particles' with dissimilar motion, bulk viscosity K_{bulk} quantifies the tendency of a fluid to resist changes in volume as it flows.
Lastly, we model the interactions with the ECM on the basal surface as a viscous drag force $b_{ecm}\mathbf{v}$ where b_{ecm} is the viscous drag coefficient. Putting this all together, the force balance equation reads:

$$\eta\bar{\nabla}^2\mathbf{v} + \frac{K_{bulk}}{2}\bar{\nabla}\cdot[\bar{\nabla}\mathbf{v}] + \gamma\alpha p^* - b_{ecm}\mathbf{v} = \mathbf{0} \tag{S7}$$

Development • Supplementary information

De-dimensionalization, boundary conditions, and solution domain

As above, we de-dimensinoalized these equations based on the dimension of variables and constants as:

$$\begin{aligned}
[\alpha] &\sim ML^{-1}T^{-2} \\
[\nabla \cdot \sigma] &\sim ML^{-2}T^{-2} \\
[\mathbf{v}] &\sim LT^{-1} \\
[b_{ecm}] &\sim ML^{-3}T^{-1} \\
[\eta], [K_{bulk}] &\sim ML^{-1}T^{-1} \\
[\mathbf{D}] &\sim T^{-1}
\end{aligned} \tag{S8}$$

from which Eq. S7 can be written in non-dimensional form as follows

$$\nabla^2 \mathbf{v}^* + \frac{K^*}{2} \nabla \cdot [\nabla \mathbf{v}^*] + \gamma \alpha^* p^* - b^*_{\text{ecm}} \mathbf{v}^* = \mathbf{0} \tag{S5}$$

where * denotes a dimensionless variable or parameter; Using the same scaling as in Eq. S3, we define the following non-dimensional parameters:

$$K^* = \frac{K_{\text{bulk}}}{\eta}, \quad \alpha^* = \frac{\alpha L}{\eta \mathrm{v}_{\text{DoT}}}, \quad b^*_{\text{ecm}} = \frac{b_{ecm} L^2}{\eta}$$

The associated boundary and initial conditions for Eq. S5 are:

$$\begin{aligned}
&\textbf{BCs:} \quad \mathbf{v}^*\Big|_{\partial\Omega} = \mathbf{0} \quad \text{except} \quad \frac{\partial \mathbf{v}^*}{\partial x}\Big|_{x=0} = 0 \ \text{ for L-R symmetry boundary} \\
&\textbf{ICs:} \quad \mathbf{v}^*\Big|_{t=0} = \mathbf{0}
\end{aligned} \tag{S9}$$

where domain $\Omega = [0, 2L] \times [-2L, 10L]$ and $\partial\Omega$ is the domain boundary. As in the transport equations above, the boundaries of the domain are sufficiently far from definitive endoderm deformations that artifacts of the boundary condition are minimized. Because the mechanics equations are also formulated and solved in a frame of reference that moves with the DoT, Q_{AP} and cell tracks were computed in a subdomain representing the definitive endoderm that moved away from the DoT with a velocity v_{DoT}. This is equivalent to solving the model in a fixed frame of reference while the DoT moves posteriorly, but because of the length scale separationbetween the domain Ω and the moving subdomain over which output metrics were computed, does not require consideration of a growing domain. Solution of the differential equations requires imposing idealized boundary conditions on the model. While even far from the forming hindgut, the endoderm continues to flow and deform, we assume that these distant movements are not governed by FGF as they exist far from the region where FGF activity is detected. This is the basis for constraining the distant anterior, lateral, and posterior boundaries to have vanishing velocities. The only local boundary is at $x = 0$, signifying the midline of the embryo. Because time lapse experiments suggest that cell movements are symmetric about this axis, it is reasonable to constrain velocities along the embryonic midline to have vanishing derivatives with respect to the x-axis on this boundary.

Numerical solution of PDEs using the Finite Element Method

Before the partial differential equations can be solved using the Finite Element Method, they must be cast in their weak form, derived by multiplying by a test function ψ, integrating over the entire domain, and resolving any second or higher order derivatives using the product rule of integration and 'off-loading' the derivatives to the test function (A. Logg et al., 2012). For the FGF transport equations, the only term with a second order derivative (i.e. which requires special handling) is the diffusive term in Eq. 3, which was treated as follows:

1. Multiply by test function ψ

$$\frac{\partial p^*}{\partial t}\psi = D^*_p \nabla^2 p^* \psi + \frac{\partial p^*}{\partial y}\psi + \beta^*_p m^* \psi - \mu^*_p p^* \psi$$

Development • Supplementary information

2. Integrate over Ω

$$\int_\Omega \frac{\partial p^*}{\partial t}\psi = D_p^* \underbrace{\int_\Omega \nabla^2 p^* \psi}_{\text{diffusion}} + \int_\Omega \frac{\partial p^*}{\partial y}\psi + \beta_p^* \int_\Omega m^* \psi - \mu_p^* \int_\Omega p^* \psi$$

3. Use Integration by parts - this part only pertains to the *diffusion* term, where the Laplacian operator (∇^2) appears,

$$\int_\Omega \nabla^2 p^* \psi = -\int_\Omega \nabla p^* \cdot \nabla \psi + \underbrace{\int_\Omega \nabla \cdot [\psi \nabla p^*]}_{0}$$

Conversion of the right-most integral above into a surface integral by way of Green's Divergence Theorem results in a term, $\int_{\partial\Omega} \psi \frac{\partial p^*}{\partial n}$, which vanishes owing to the no flux boundary conditions, i.e. $\frac{\partial p^*}{\partial n} = 0$ on $\partial\Omega$.

4. Discretize time derivative operator using first order backward Euler scheme:

$$\int_\Omega \frac{p^* - {p^*}_{(t-1)}}{\Delta t}\psi = -D_p^* \int_\Omega \nabla p^* \cdot \nabla \psi + \int_\Omega \frac{\partial p^*}{\partial y}\psi + \beta_p^* \int_\Omega m^* \psi - \mu_p^* \int_\Omega p^* \psi$$

where ${p^*}_{(t-1)}$ is the solution from the previous time step (starting with the initial condition).

Following a similar approach for the tissue mechanics model as outlined for the FGF transport model above, the corresponding weak forms were directly inputted and solved in the python-based, open-source package `FEniCS`.

Model assumptions, limitations, and future considerations

Although the model successfully captures aspects of normal hindgut development as well as responses to perturbations that influence contractility and FGF sensing in the endoderm (Fig. 6, Supplemental Movie 2), there are several important model assumptions and limitations that must be considered in the direct context of the methods, described briefly in the main text and expanded upon here.

Establishment of the FGF gradient

For simplicity, we assumed in the present work that FGF ligand concentration in the presomitic mesoderm varies linearly with activity of the Dusp6 reporter within the neighboring endoderm. This is admittedly an indirect approach for estimating FGF protein concentrations in the extracellular space, implicitly assuming that there is a proportionality between reporter activity and FGF protein concentration. This approach resulted in an estimation of FGF ligand diffusivity as $\sim$12.5 μm^2/s. This agrees reasonably well with direct measurements of single FGF8 molecules performed in gastrulating zebrafish embryos, where a subpopulation of molecules interacting with extracellular heparan sulfate proteoglycans were found to diffuse at $\sim$4 μm^2/s (compared to the faster diffusion of $\sim$50 μm^2/s measured in the remaining population) (Yu et al., 2009). Similar diffusion coefficients have been measured for morphogens in various contexts including Dpp in the *Drosophila* wing disc (Zhou et al., 2012), Wnt3a in the *Xenopus* gastrula (Takada et al., 2018), and TGF-β family ligands in zebrafish (M¨uller et al., 2012). This broad agreement suggests that equating Dusp6 reporter activity with FGF ligand concentration may be a reasonable simplification. Further, pseudo-space analyses of published single-cell RNA Seq datasets (Ibarra-Soria et al., 2018) in the mouse endoderm suggest that FGF receptors are uniformly expressed along the antero-posterior axis (Fig. S6). Looking to other potential readouts of FGF activity, we previously compared the DUSP6 reporter with immunostaining for diphospho-ERK1/2, finding that each produced similar results, such that FGF activity decreases exponentially, reaching background levels by approximately 900 μm from the caudal intestinal portal (Nerurkar et al., 2019). Motivated by our prior work, the present study is squarely focused on FGF signaling as it relates to hindgut elongation. However, the long-range gradient of FGF proteins in the

Development • Supplementary information

posterior embryo parallels a Wnt gradient, and is anti-parallel to a reciprocal anterior-to-posterior gradient of retinoic acid. Mutual antagonism between retinoic acid and FGF signaling pathways has been observed in many contexts (Aulehla & Pourqui´e, 2010; Bayha et al., 2009; Lin et al., 2010), but it has not yet been studied whether retinoic acid has any influence on endoderm cell movements during gut tube morphogenesis.

Linking FGF activity to downstream acto-myosin contractility

Despite the widespread evidence that FGF signaling coordinates cell migration and other acto-myosin dependent cell behavior in many contexts (B´enaz´eraf et al., 2010; Ernst et al., 2012; Harding & Nechiporuk, 2012; Sai & Ladher, 2008; A. Sato et al., 2011), very little is known regarding the molecular effectors downstream of FGF that are involved in regulating acto-mysoin activity. To focus the present study on how upstream transport properties and downstream tissue mechanics are integrated despite this, we modeled the relationship between FGF protein and contractility as simply as possible: the two were treated as linearly related by a proportionality constant α^*. Treating cell shape as an approximate readout of active tension, we observed that cell shape is linearly correlated with the inverse of FGF activity along medio-lateral and antero-poserior axes (Fig. S4), suggesting that a proportional relationship between contractility and FGF concentration may be appropriate within the physiologic range of protein concentrations. From prior work, we known that FGF proteins acts within minutes through transcription-independent mechanisms to increase endoderm tension via RhoA-dependent mechanisms, and that perturbations that increase or decrease FGF activity coordinately increase or decrease RhoA activation and tissue-scale tension (Nerurkar et al., 2019). Together, these findings suggest that even if the relationship between FGF protein and active stress may not be entirely proportional, it is likely still relatively simple. Modeling it as such allows us to minimize the number of free parameters in the model, and to focus upstream and downstream of this on interactions between transport phenomena and tissue mechanics. Nonetheless, it is important to recognize the growing appreciation for intracellular signaling dynamics, rather than stable protein levels or modifications, as important regulators of downstream cell behaviors (Simsek et al., 2023; Simsek & ¨Ozbudak, 2018). These dynamics can be quite complex and highly nonlinear (Aldridge et al., 2006; Lee et al., 2022; Prahl & Odde, 2018). Of particular note are the recent studies focused on dynamics of ERK activity (De La Cova et al., 2017; Deathridge et al., 2019). ERK is a downstream readout of FGF signaling, among other pathways. Oscillations of ERK activity have been observed in several contexts, including zebrafish wound healing (De Simone et al., 2021) and somitogenesis (Simsek & ¨Ozbudak, 2018), and mouse ESCs (Raina et al., 2022), among others. Further, optogenetic manipulation of the frequency of ERK activity has been shown to regulate a range of events during development, including ventral furrow formation in Drosophila (Johnson & Toettcher, 2019) and somite boundary formation in zebrafish (Simsek et al., 2023) and mice (Niwa et al., 2007). Of additional note, collective cell movements of MDCK cells in vitro have been shown to be regulated by ERK activity waves as well (Aoki et al., 2017). Whether oscillatory ERK activity or related feedback mechanism are involved in potentiation of FGF protein effects on acto-myosin activity is not yet known, but will be important to consider in future experimental studies on amniote hindgut morphogenesis.

Mechanical properties of the endoderm

From the mechanics standpoint, studies in the neighboring presomitic mesoderm suggest that FGF increases tissue 'fluidity' (Lawton et al., 2013), and a gradient in fluid-to-solid like physical properties has been identified along the antero-posterior axis in the presomitic mesoderm (Mongera et al., 2018). Physical properties of the endoderm, however, have not been characterized, and heterogeneity in such properties has therefore not been observed. Cell-cell adhesion and basement membrane composition appear similar throughout the endoderm (Nerurkar et al., 2019), suggesting that any positional differences in viscosity of the cells or basement membrane may be minimal. In addition, removal of cell contractility via disruption of the actin cytoskeleton results in a loss of the tensional gradient normally observed in the posterior embryo, suggesting that differential growth, stiffness, and other contributions to the tension gradient are negligible.

ECM plays a critical role in guiding (Araya et al., 2016; Kyprianou et al., 2020) and resisting (Crest et al., 2017; Danesin et al., 2021) cell behaviors to coordinate morphogenesis. In the present study, the basement membrane was modeled as a viscous drag resisting cell movements in the endoderm. This was based on prior studies in the epiblast (Zamir et al., 2008), presomitic mesoderm (B´enaz´eraf et al., 2010), and precardiac mesoderm and foregut endoderm (Aleksandrova et al., 2015), which each suggest that the ECM in the early avian embryo is highly dynamic and tends to passively flow with cells as they reorganize. Further, disruption of contractility in the posterior endoderm during hindgut formation does not cause a full reversal of cell movements, suggesting that

Development • Supplementary information

the basement membrane is not storing elastic energy as the endoderm flows posteriorly (Nerurkar et al., 2019). Together, these observatoions suggest that the ECM may resist endoderm movement more as a drag force or dashpot rather than as a spring that is progressively stretched as endoderm moves. Nonetheless, based on growing evidence of an instructive role played by the ECM during morphogenesis (Crest et al., 2017; S. E. Harrison et al., 2018), and improved tools for live imaging of extracellular matrix in vivo (Fischer et al., 2023; Y. Sato et al., 2017), moving forward it will be important to understand how the basement membrane participates in gut tube formation.

When solving partial differential equations, it is necessary to impose conditions that effectively constrain the solution to known quantities on the margins of the domain. This poses a common challenge in modeling of embryonic development, where boundaries of the embryo are complex, highly dynamic, and often poorly defined from the biophysical standpoint. As a result, commonly used, mathematically tractable boundary conditions reflect highly idealized scenarios that essentially never occur in a developing embryo (e.g. perfect adsorption of protein or zero displacement or velocity). In an effort to minimize potential artifacts from the imposed boundary conditions, we chose a common approach of placing the boundaries very far from the region of interest, which serves to insulate the solution locally from artificially limiting boundary constraints. It was assumed that endoderm velocity vanishes at the anterior, lateral, and posterior boundaries. While this is an oversimplifcation, making the domain size an order of magnitude larger than the range of FGF activity is consistent with observations in the embryo, where FGF activity drops to undetectable levels well before reaching the anterior intestinal portal or the lateral boundary between definitive endoderm and hypoblast cells (Nerurkar et al., 2019). One potential challenge to address in future work relates to the posterior boundary of the definitive endoderm during hindgut formation. In the embryo, as endoderm cells move posteriorly past the caudal intestinal portal (Nerurkar et al., 2019), they drive folding of the endoderm and inversion along the dorso-ventral axis, something that cannot be well-captured in a 2-D model of the coronal plane. It may first be necessary to understand descriptively how cell movements and forces are organized in the elongating hindgut, which as an internal structure is not amenable to live imaging.

Finally, the present study only considers chemo-mechanical coupling uni-directionally, with FGF concentration influencing tissue deformations, but not vice versa. This was largely based on previous evidence that while *Fgf4* and *Fgf8* are expressed within the posterior-most endoderm, the broader expression pattern of these genes in the neighboring presomitic mesoderm aligns more closely with the extent of DUSP6 reporter activity, diphospho-ERK staining, and FGF target gene expression in the endoderm (Nerurkar et al., 2019). Therefore, the primary source of protein guiding tissue deformations movements is assumed to be mesodermal, and therefore is external to the endoderm. Nonetheless, it is possible that endoderm flows redistribute FGF proteins, and that the resulting bi-directional coupling between biochemical and mechanical properties may be important to consider as well.

Code Availability

The code to solve the equations of the chemo-mechanical model presented above is deposited in the form of an executable 'notebook' (Github). Please consult the official FEniCS website for installation instructions.

Development • Supplementary information

References

A. Logg, G. N. Wells, & K.-A. Mardal A. (2012). *Automated Solution of Differential Equations by the Finite Element Method*. Springer. https://doi.org/10.1007/978-3-642-23099-8

Aldridge, B. B., Burke, J. M., Lauffenburger, D. A., & Sorger, P. K. (2006). Physicochemical modelling of cell signalling pathways. *Nature Cell Biology*, *8*(11), 1195–1203. https://doi.org/10.1038/ncb1497

Aleksandrova, A., Czirok, A., Kosa, E., Galkin, O., Cheuvront, T. J., & Rongish, B. J. (2015). The endoderm and myocardium join forces to drive early heart tube assembly. *Developmental Biology*, *404*(1), 40–54. https://doi.org/10.1016/j.ydbio.2015.04.016

Aoki, K., Kondo, Y., Naoki, H., Hiratsuka, T., Itoh, R. E., & Matsuda, M. (2017). Propagating Wave of ERK Activation Orients Collective Cell Migration. *Developmental Cell*, *43*(3), 305–317.e5. https://doi.org/10.1016/j.devcel.2017.10.016

Araya, C., Carmona-Fontaine, C., & Clarke, J. D. (2016). Extracellular matrix couples the convergence movements of mesoderm and neural plate during the early stages of neurulation: ECM is Required During Zebrafish Neurulation. *Developmental Dynamics*, *245*(5), 580–589. https://doi.org/10.1002/dvdy.24401

Aulehla, A., & Pourquié, O. (2010). Signaling gradients during paraxial mesoderm development. *Cold Spring Harbor perspectives in biology*, *2*(2), a000869. https://doi.org/10.1101/cshperspect.a000869

Baker, D. E. (2002). A Geometric Method for Determining Shape of Bird Eggs (J. Brawn, Ed.). *The Auk*, *119*(4), 1179–1186. https://doi.org/10.1093/auk/119.4.1179

Baker, R. E., Schnell, S., & Maini, P. K. (2008). Mathematical Models for Somite Formation [ISBN: 0123742536]. *Current Topics in Developmental Biology*, *81*(07), 183–203. https://doi.org/10.1016/S0070-2153(07)81006-4

Bayha, E., Jørgensen, M. C., Serup, P., & Grapin-Botton, A. (2009). Retinoic acid signaling organizes endodermal organ specification along the entire antero-posterior axis [ISBN: 1932-6203 (Electronic)]. *PLoS ONE*, *4*(6). https://doi.org/10.1371/journal.pone.0005845

Bénazéraf, B., Francois, P., Baker, R. E., Denans, N., Little, C. D., & Pourquié, O. (2010). A random cell motility gradient downstream of FGF controls elongation of an amniote embryo. *Nature*, *466*(7303), 248–252. https://doi.org/brisco

Crest, J., Diz-Muñoz, A., Chen, D. Y., Fletcher, D. A., & Bilder, D. (2017). Organ sculpting by patterned extracellular matrix stiffness. *eLife*, *6*. https://doi.org/10.7554/eLife.24958

Danesin, C., Ferreira, M. A., Degond, P., & Theveneau, E. (2021). Anteroposterior elongation of the chicken anterior trunk neural tube is hindered by interaction with its surrounding tissues. *Cells & Development*, *168*, 203723. https://doi.org/10.1016/j.cdev.2021.203723

De La Cova, C., Townley, R., Regot, S., & Greenwald, I. (2017). A Real-Time Biosensor for ERK Activity Reveals Signaling Dynamics during C. elegans Cell Fate Specification. *Developmental Cell*, *42*(5), 542–553.e4. https://doi.org/10.1016/j.devcel.2017.07.014

De Simone, A., Evanitsky, M. N., Hayden, L., Cox, B. D., Wang, J., Tornini, V. A., Ou, J., Chao, A., Poss, K. D., & Di Talia, S. (2021). Control of osteoblast regeneration by a train of Erk activity waves. *Nature*, *590*(7844), 129–133. https://doi.org/10.1038/s41586-020-03085-8

Deathridge, J., Antolović, V., Parsons, M., & Chubb, J. R. (2019). Live imaging of ERK signaling dynamics in differentiating mouse embryonic stem cells. *Development*, dev.172940. https://doi.org/10.1242/dev.172940

Ernst, S., Liu, K., Agarwala, S., Moratscheck, N., Avci, M. E., Dalle Nogare, D., Chitnis, A. B., Ronneberger, O., & Lecaudey, V. (2012). Shroom3 is required downstream of FGF signalling to mediate proneuromast assembly in zebrafish. *Development (Cambridge, England)*, *139*(24), 4571–81. https://doi.org/10.1242/dev.083253

Fischer, A., Correa-Gallegos, D., Wannemacher, J., Christ, S., Machens, H.-G., & Rinkevich, Y. (2023). In vivo fluorescent labeling and tracking of extracellular matrix. *Nature Protocols*. https://doi.org/10.1038/s41596-023-00867-y

Harding, M. J., & Nechiporuk, a. V. (2012). Fgfr-Ras-MAPK signaling is required for apical constriction via apical positioning of Rho-associated kinase during mechanosensory organ formation. *Development*, *139*(18), 3467–3467. https://doi.org/10.1242/dev.087999

Harrison, N. C., del Corral, D. R., & Vasiev, B. (2011). Coordination of cell differentiation and migration in mathematical models of caudal embryonic axis extension [ISBN: 1932-6203]. *PLoS ONE*, *6*(7). https://doi.org/10.1371/journal.pone.0022700

Harrison, S. E., Sozen, B., & Zernicka-Goetz, M. (2018). In vitro generation of mouse polarized embryo-like structures from embryonic and trophoblast stem cells. *Nature Protocols*, *13*(7), 1586–1602. https://doi.org/10.1038/s41596-018-0005-x

Development • Supplementary information

Ibarra-Soria, X., Jawaid, W., Pijuan-Sala, B., Ladopoulos, V., Scialdone, A., Jörg, D. J., Tyser, R. C. V., Calero-Nieto, F. J., Mulas, C., Nichols, J., Vallier, L., Srinivas, S., Simons, B. D., Göttgens, B., & Marioni, J. C. (2018). Defining murine organogenesis at single-cell resolution reveals a role for the leukotriene pathway in regulating blood progenitor formation. *Nature Cell Biology*, *20*(2), 127–134. https://doi.org/10.1038/s41556-017-0013-z

Johnson, H. E., & Toettcher, J. E. (2019). Signaling Dynamics Control Cell Fate in the Early Drosophila Embryo. *Developmental Cell*, *48*(3), 361–370.e3. https://doi.org/10.1016/j.devcel.2019.01.009

Kyprianou, C., Christodoulou, N., Hamilton, R. S., Nahaboo, W., Boomgaard, D. S., Amadei, G., Migeotte, I., & Zernicka-Goetz, M. (2020). Basement membrane remodelling regulates mouse embryogenesis. *Nature*, *582*(7811), 253–258. https://doi.org/10.1038/s41586-020-2264-2

Lawton, A. K., Nandi, A., Stulberg, M. J., Dray, N., Sneddon, M. W., Pontius, W., Emonet, T., & Holley, S. A. (2013). Regulated tissue fluidity steers zebrafish body elongation. *Development*, *140*(3), 573–582. https://doi.org/10.1242/dev.090381

Lee, S. H., Hou, J. C., Hamidzadeh, A., Yousafzai, M. S., Ajeti, V., Chang, H., Odde, D. J., Murrell, M., & Levchenko, A. (2022). A molecular clock controls periodically driven cell migration in confined spaces. *Cell Systems*, *13*(7), 514–529.e10. https://doi.org/10.1016/j.cels.2022.05.005

Lin, S.-C., Dollé, P., Ryckebüsch, L., Noseda, M., Zaffran, S., Schneider, M. D., & Niederreither, K. (2010). Endogenous retinoic acid regulates cardiac progenitor differentiation. *Proceedings of the National Academy of Sciences of the United States of America*, *107*(20), 9234–9239. https://doi.org/10.1073/pnas.0910430107

Mongera, A., Rowghanian, P., Gustafson, H. J., Shelton, E., Kealhofer, D. A., Carn, E. K., Serwane, F., Lucio, A. A., Giammona, J., & Campàs, O. (2018). A fluid-to-solid jamming transition underlies vertebrate body axis elongation [Number: 7723 Publisher: Nature Publishing Group]. *Nature*, *561*(7723), 401–405. https://doi.org/10.1038/s41586-018-0479-2

Müller, P., Rogers, K. W., Jordan, B. M., Lee, J. S., Robson, D., Ramanathan, S., & Schier, A. F. (2012). Differential Diffusivity of Nodal and Lefty Underlies a Reaction-Diffusion Patterning System. *Science*, *336*(6082), 721–724. https://doi.org/10.1126/science.1221920

Nerurkar, N. L., Lee, C. H., Mahadevan, L., & Tabin, C. J. (2019). Molecular control of macroscopic forces drives formation of the vertebrate hindgut [Publisher: Nature Publishing Group]. *Nature*, *565*(7740), 480–484. https://doi.org/10.1038/s41586-018-0865-9

Niwa, Y., Masamizu, Y., Liu, T., Nakayama, R., Deng, C.-X., & Kageyama, R. (2007). The Initiation and Propagation of Hes7 Oscillation Are Cooperatively Regulated by Fgf and Notch Signaling in the Somite Segmentation Clock. *Developmental Cell*, *13*(2), 298–304. https://doi.org/10.1016/j.devcel.2007.07.013

Prahl, L. S., & Odde, D. J. (2018). Modeling Cell Migration Mechanics [Series Title: Advances in Experimental Medicine and Biology]. In C. Dong, N. Zahir, & K. Konstantopoulos (Eds.), *Biomechanics in Oncology* (pp. 159–187). Springer International Publishing. https://doi.org/10.1007/978-3-319-95294-9_9

Raina, D., Fabris, F., Morelli, L. G., & Schröter, C. (2022). Intermittent ERK oscillations downstream of FGF in mouse embryonic stem cells. *Development*, *149*(4), dev199710. https://doi.org/10.1242/dev.199710

Regev, I., Guevorkian, K., Gupta, A., Pourquié, O., & Mahadevan, L. (2022). Rectified random cell motility as a mechanism for embryo elongation. *Development*, *149*(6), dev199423. https://doi.org/10.1242/dev.199423

Sai, X., & Ladher, R. K. (2008). FGF Signaling Regulates Cytoskeletal Remodeling during Epithelial Morphogenesis. *Current Biology*, *18*(13), 976–981. https://doi.org/10.1016/j.cub.2008.05.049

Sato, A., Scholl, A. M., Kuhn, E., Stadt, H. A., Decker, J. R., Pegram, K., Hutson, M. R., & Kirby, M. L. (2011). FGF8 signaling is chemotactic for cardiac neural crest cells. *Developmental Biology*, *354*(1), 18–30. https://doi.org/10.1016/j.ydbio.2011.03.010

Sato, Y., Nagatoshi, K., Hamano, A., Imamura, Y., Huss, D., Uchida, S., & Lansford, R. (2017). Basal filopodia and vascular mechanical stress organize fibronectin into pillars bridging the mesoderm-endoderm gap. *Development*, *144*(2), 281–291. https://doi.org/10.1242/dev.141259

Serra, M., Nájera, G. S., Chuai, M., Spandan, V., Weijer, C. J., & Mahadevan, L. (2021). A mechanochemical model recapitulates distinct vertebrate gastrulation modes. *bioRxiv*, 2021.10.03.462928. https://doi.org/10.1101/2021.10.03.462928

Simsek, M. F., Chandel, A. S., Saparov, D., Zinani, O. Q. H., Clason, N., & Özbudak, E. M. (2023). Periodic inhibition of Erk activity drives sequential somite segmentation. *Nature*, *613*(7942), 153–159. https://doi.org/10.1038/s41586-022-05527-x

Simsek, M. F., & Özbudak, E. M. (2018). Spatial Fold Change of FGF Signaling Encodes Positional Information for Segmental Determination in Zebrafish. *Cell Reports*, *24*(1), 66–78.e8. https://doi.org/10.1016/j.celrep.2018.06.023

Takada, R., Mii, Y., Krayukhina, E., Maruyama, Y., Mio, K., Sasaki, Y., Shinkawa, T., Pack, C.-G., Sako, Y., Sato, C., Uchiyama, S., & Takada, S. (2018). Assembly of protein complexes restricts diffusion of Wnt3a proteins. *Communications Biology*, *1*(1), 165. https://doi.org/10.1038/s42003-018-0172-x

Xiong, F., Ma, W., Bénazéraf, B., Mahadevan, L., & Pourquié, O. (2020). Mechanical Coupling Coordinates the Co-elongation of Axial and Paraxial Tissues in Avian Embryos. *Developmental Cell*, *55*(3), 354–366.e5. https://doi.org/10.1016/j.devcel.2020.08.007

Yu, S. R., Burkhardt, M., Nowak, M., Ries, J., Petrásek, Z., Scholpp, S., Schwille, P., & Brand, M. (2009). Fgf8 morphogen gradient forms by a source-sink mechanism with freely diffusing molecules. *Nature*, *461*(7263), 533–536. https://doi.org/10.1038/nature08391

Zamir, E. a., Rongish, B. J., & Little, C. D. (2008). The ECM moves during primitive streak formation - Computation of ECM versus cellular motion [ISBN: 1545-7885 (Electronic)]. *PLoS Biology*, *6*(10), 2163–2171. https://doi.org/10.1371/journal.pbio.0060247

Zhou, S., Lo, W.-C., Suhalim, J. L., Digman, M. A., Gratton, E., Nie, Q., & Lander, A. D. (2012). Free Extracellular Diffusion Creates the Dpp Morphogen Gradient of the Drosophila Wing Disc. *Current Biology*, *22*(8), 668–675. https://doi.org/10.1016/j.cub.2012.02.065

Development • Supplementary information
